# Including Volume Effects in Biological Treatment Plan Optimization for Carbon Ion Therapy: Generalized Equivalent Uniform Dose-Based Objective in TRiP98

**DOI:** 10.3389/fonc.2022.826414

**Published:** 2022-03-21

**Authors:** Marco Battestini, Marco Schwarz, Michael Krämer, Emanuele Scifoni

**Affiliations:** ^1^ Department of Physics, University of Trento, Trento, Italy; ^2^ Trento Institute for Fundamental Physics and Applications (TIFPA), Istituto Nazionale di Fisica Nucleare (INFN), Trento, Italy; ^3^ Trento Proton Therapy Center, Azienda Provinciale per i Servizi Sanitari (APSS), Trento, Italy; ^4^ Biophysics Department, GSI - Helmholtzzentrum für Schwerionenforschung, Darmstadt, Germany

**Keywords:** volume effect in radiotherapy, generalized equivalent uniform dose (gEUD), TRiP98, carbon ion therapy, treatment plan optimization, biological treatment planning, normal tissue complication probability (NTCP)

## Abstract

We describe a way to include biologically based objectives in plan optimization specific for carbon ion therapy, beyond the standard voxel-dose-based criteria already implemented in TRiP98, research planning software for ion beams. The aim is to account for volume effects—tissue architecture-dependent response to damage—in the optimization procedure, using the concept of generalized equivalent uniform dose (gEUD), which is an expression to convert a heterogeneous dose distribution (e.g., in an organ at risk (OAR)) into a uniform dose associated with the same biological effect. Moreover, gEUD is closely related to normal tissue complication probability (NTCP). The multi-field optimization problem here takes also into account the relative biological effectiveness (RBE), which in the case of ion beams is not factorizable and introduces strong non-linearity. We implemented the gEUD-based optimization in TRiP98, allowing us to control the whole dose–volume histogram (DVH) shape of OAR with a single objective by adjusting the prescribed *gEUD*_0_ and the volume effect parameter *a*, reducing the volume receiving dose levels close to mean dose when *a* = 1 (large volume effect) while close to maximum dose for *a* >> 1 (small volume effect), depending on the organ type considered. We studied the role of *gEUD*_0_ and *a* in the optimization, and we compared voxel-dose-based and gEUD-based optimization in chordoma cases with different anatomies. In particular, for a plan containing multiple OARs, we obtained the same target coverage and similar DVHs for OARs with a small volume effect while decreasing the mean dose received by the proximal parotid, thus reducing its NTCP by a factor of 2.5. Further investigations are done for this plan, considering also the distal parotid gland, obtaining a NTCP reduction by a factor of 1.9 for the proximal and 2.9 for the distal one. In conclusion, this novel optimization method can be applied to different OARs, but it achieves the largest improvement for organs whose volume effect is larger. This allows TRiP98 to perform a double level of biologically driven optimization for ion beams, including at the same time RBE-weighted dose and volume effects in inverse planning. An outlook is presented on the possible extension of this method to the target.

## 1 Introduction

In the last decades, the physical and radiobiological properties of charged particles have been extensively studied, as an alternative to the traditional photons in radiation therapy (1). It was in particular emphasized that ion beams heavier than protons combine both physical and biological advantages (2). Among these particles, carbon ions have now reached clinical use in a dozen of treatment centers around the world, and typically, they are used for tumors that are inoperable or resistant to traditional treatments (3). Their use is presently still limited, primarily because of economic and logistical reasons, but also due to greater difficulty in characterizing and modeling the physics and radiobiological effects of ion beams on the biological tissues (4).

In state-of-the-art centers like HIT (Heidelberg), CNAO (Pavia), MED-Austron (Wiener Neustadt), or HIMAC (Chiba), the dose is delivered in a so-called “raster scanning mode”, which allows optimal flexibility for improved tumor three-dimensional dose shaping and sparing of healthy tissues. The scanning parameters and the resulting dose distribution are obtained using dedicated treatment planning systems (TPSs) accounting for both physical and biological effects of the particles. TRiP98 (TReatment plannIng for Particles), the GSI (Darmstadt) research planning software for ion beams (5, 6), was the first TPS of this type and served as a reference for the following ones. This software allows to determine the optimal 3D biological dose distribution for a specific patient, imposing a uniform dose for the tumor and a maximum dose objective for critical structures. TRiP98 was used clinically during the GSI therapy pilot project, which started in 1997 in collaboration with DKFZ (Heidelberg), the University Clinic Heidelberg, and the FZ Rossendorf (Dresden), when 440 patients have been treated with carbon ions in an 11-year span, in particular with head and neck cancers (7). After that, it has been extensively used and expanded until now, as an advanced research tool for biological treatment planning with ion beams, including among others multiple-field optimization (8), advanced relative biological effectiveness (RBE)-weighted dose algorithms (9), oxygen enhancement ratio (OER)-driven optimization (10), helium and oxygen beams characterization (11, 12), and multiple-ion optimization (13). Despite all these advanced implementations, the volume effect was never included in the TRiP98 optimization, which is only based on a single dose value to date, i.e., the prescribed dose for target and a maximum dose for organs at risk (OARs).

Organs and tissues have a biological architecture, allowing them to perform specific functions. Such architecture has consequences also on the response of healthy organs to radiation, which is more complex than the response of an ensemble of cells that behave independently from one another. A key aspect determining the response of organized biological tissue to ionizing radiation is the so-called volume effect (14), which can be qualitatively described as the capability of an organ to compensate the radiation damage to part of it as long as the rest of the organ is sufficiently spared. There have been different proposals in the literature on a quantitative description of the volume effect. At the moment, one of the most commonly used is the generalized equivalent uniform dose (gEUD) proposed by Niemierko (15), which is an expression based on a power law dose–effect relation converting a heterogeneous dose distribution into a homogeneous dose distribution with the same biological effect.

There is a strong rationale for including volume effects in treatment planning optimization, in particular for OARs showing large volume effects, such as the lung, liver or parotid glands. Furthermore, the inclusion of the volume effect is a stepping stone towards the definition of cost functions that directly optimize the most clinically relevant parameters concerning healthy tissues, i.e., the normal tissue complication probability (NTCP).

Such an approach, which goes in the direction of what is called biologically oriented treatment planning, was tested for photon radiotherapy in a number of studies (16–18), investigating different approaches to extend the objective function for efficiently minimizing the gEUD. Over the past 10 years, the availability of gEUD-based optimization for photon radiotherapy in clinical practice has significantly increased. The use of gEUD in plan optimization has been addressed already in 2012 by Allen et al. (19). Only recently a single attempt to translate it for carbon ion therapy was performed based on different formulations for the equivalent uniform dose (EUD) (20). The latter case in fact is complicated by the additional biological level involved in a carbon plan optimization, namely, the RBE, a strongly non-linear effect.

The purpose of this work is to include objectives related to the volume effect in plan optimization for carbon ion therapy, in addition to the standard voxel-dose-based criteria already implemented in TRiP98. This approach should allow to optimize the dose in a different way according to the type of organ considered, attempting to improve the sparing of critical structures and therefore reducing the probability of complications.

This paper is organized as follows: we first describe the link between tissue architecture and normal tissue response to radiation, and we present an expression for gEUD. We then introduce the optimization problem in TRiP98; in particular, we define the cost function, we describe how the dose is calculated, and we present the optimization algorithms used in this study. We discuss in detail the novel optimization method based on the gEUD implemented in TRiP98 for OARs, and we show some treatment planning examples, comparing the new gEUD-based approach with the standard voxel-dose-based method. Finally, we discuss possible additional implementations for the optimization of the target and developments towards direct NTCP-based optimization.

## 2 Materials and Methods

### 2.1 Tissue Architecture and Volume Effect

When complex biological systems are considered, like tissues or organs, cells are organized in structures that are often called functional sub-units (FSUs), which may also be visible at the morphological level (e.g., in lung alveoli or kidney nephrons). The volume effect can be interpreted as a consequence of the fact that FSUs can be organized in different ways in different organs (14). For instance, a small volume effect (i.e., the fact that the organ damage is determined by the maximum dose, even if delivered to a very small portion of the organ itself) is the consequence of FSUs organized in form of a chain. In this case, it is sufficient that a single element of the chain breaks down for the chain not to exist anymore. This is the reason why complications associated with a small volume effect are also referred to as “serial”. In the case of complications with a large volume effect (e.g., radiation pneumonitis), where the mean dose is the parameter that best correlates with the outcome, the FSUs are instead organized as threads of a rope. In this case, the rope is still functional as long as a sufficient number of threads are working, thus the name of “parallel” complication.

The “parallel” and “serial” behaviors are simplifications. In reality, each organ, and even each complication for the same organ, will have its own specific volume effect, which can be anywhere between a purely serial and purely parallel behavior.

#### 2.1.1 Generalized Equivalent Uniform Dose

The gEUD is an expression to convert a heterogeneous dose distribution into a uniform dose associated with the same biological effect (15); the conversion is based on a power law:


(1) $$gEUD\;[Gy]={\left(\frac{1}{M}\sum_{i=1}^{M}D_{i}^{a}\right)}^{\frac{1}{a}}$$

where *D_i_* is the dose associated with the voxel *i*, *M* is the number of voxels of the anatomical structure considered, and *a* is the parameter quantifies the volume effect of the organ/tissue considered, and it is specific to each biological structure (or each type of complication). For *a* → −∞ ⇒ *gEUD* → *D_min_*, for *a* → +∞ ⇒ *g*EUD → *D_max_*, and *a*=1 ⇒ *gEUD* = *D_mean_*. This phenomenological description can be applied to both tumors (*a* < 0) and normal tissues (*a* < 0).

In addition to representing a more realistic description of the dose–effect relation for healthy tissues, from an optimization perspective, the use of gEUD has the advantage of providing a single metric able to control the volume irradiated from 0 to maximum dose, while dose–volume histogram (DVH)-based optimization considers only one dose value per DVH point.

The benefits of the using gEUD in the optimization of treatment plans have been investigated in the case of photon therapy, for different TPS and using different types of optimization algorithms [see e.g., Wu et al. (16), Schwarz et al. (18), and Fogliata et al. (21)].

The goal of this kind of optimization is to achieve a reduction in dose to the OAR focusing on the dose range that matters the most for that specific organ or complication. For instance, in the case of organs where the probability of complication is related to the mean dose, the setting *a* = 1 implies that the optimizer will have the same incentive in achieving dose reduction anywhere between 0 and maximum dose. On the other hand, in the case of small volume effects, by setting *a* >> 1, the *gEUD* will be largely determined by the DVH shape at high doses, thus creating an incentive for the optimizer to reduce the dose mostly in that dose range.

#### 2.1.2 Normal Tissue Complication Probability

The probability of encountering a radiotherapy side effect is typically quantified *via* NTCP models. Several NTCP models exist, and the so-called Lyman–Kutcher–Burman (LKB) model (22, 23) is the most commonly used so far. An additional advantage of the LKB model in the context of our work is that its formulation is consistent with the gEUD expression, and it is therefore possible to use it as a phenomenological description of the dose–effect relation for an OAR. In the LKB formulation, the NTCP is defined as


(2) $$NTCP\;(u)=\frac{1}{\sqrt{2\pi }}\int_{-\infty }^{u}e^{-\frac{t^{2}}{2}}dt$$

where


(3) $$u=\frac{gEUD-TD_{50}}{m\cdot TD_{50}}$$

*TD_50_* is the whole organ dose corresponding to 50% complication probability and *m* is the slope of the dose–response curve at *TD_50_*. Therefore, an organ that receives a heterogeneous dose, described by a DVH, has the same NTCP as if it was irradiated with a uniform dose equal to *gEUD*.

### 2.2 Optimization in TRiP98

**Figure 1:**
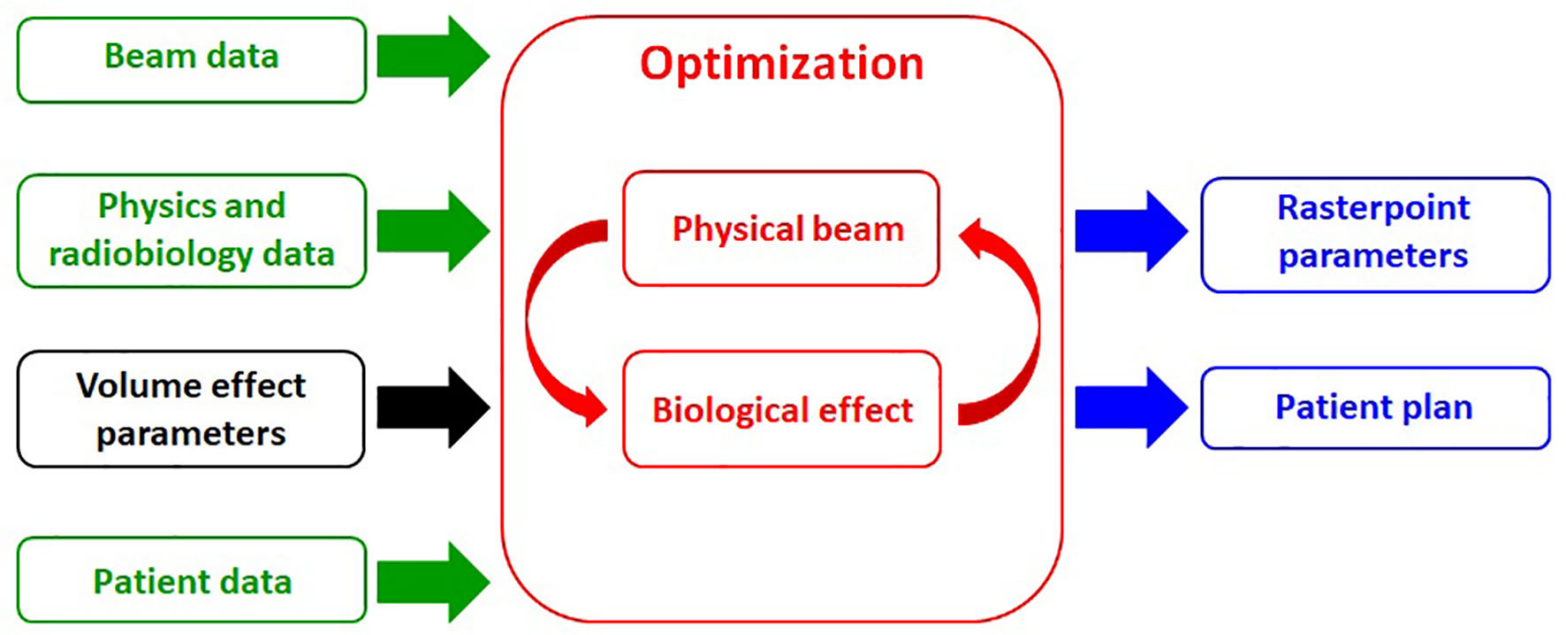
Simplified flowchart structure of TRiP98: input data (green boxes), including the extension developed in this work, i.e., the implementation of a generalized equivalent uniform dose (gEUD)-based objective in the optimization procedure (black box); optimization task (red boxes); output data (blue boxes).

A radiotherapy treatment plan for a patient is a calculated dose distribution that achieves a satisfactory balance between the tumor control probability and the sparing of healthy tissues. In actively scanned particle therapy, the dose is usually delivered using a raster scanning system, which maximizes the degrees of freedom available in dose delivery, and as a consequence in dose shaping. In order to generate a treatment plan, a computational engine like TRiP98 is used: this allows to optimize the vector of particle numbers ${\vec{N}}_{opt}$ for all rasterpoints from all fields in order to obtain a 3D dose distribution that respects the objectives imposed (plan optimization), taking into account patient data (CT images, volume of interest (VOI) contours, the prescribed and maximum doses for each VOI, etc.), beam data (number of fields, ion species, available energies, etc.), and also physics and radiobiology data (depth-dose distributions, particle energy spectra, RBE, etc.). The optimization task produces in the output the scanner parameters (beam energies, particle fluences and positions) and the patient plan (DVH, dose distribution, etc.). The crucial part of the production of an acceptable treatment plan is the optimization task. The TRiP98 structure is schematized in **Figure 1**.

#### 2.2.1 Objective Function

The starting point of the optimization procedure is the definition of a cost function, which formalizes the treatment goals in a mathematical expression. In a clinically realistic case, these objectives are in conflict with each other, and the final dose distribution is the best achievable compromise given such objectives. In TRiP98, the available objectives are uniform dose to the target and an upper dose value for each OAR (5, 6). The TRiP98 cost function is defined as follows:


(4) $$\chi_{D_{pre}/D_{\max}}^{2}(\vec{N})={\left(w_{T}\right)}^{2}\sum_{i=1}^{M_{T}}\frac{{\left(D_{pre}-D_{i}(\vec{N})\right)}^{2}}{{\left(\Delta D_{pre}\right)}^{2}}+{\left(w_{OAR}\right)}^{2}\;\sum_{i=1}^{M_{OAR}}\frac{{\left(D_{\max}-D_{i}(\vec{N})\right)}^{2}}{{\left(\Delta D_{\max}\right)}^{2}}\;\theta_{D_{\max}}$$

where $\vec{N}$ is the vector of particle numbers; *M_T_* and *M_OAR_* are the total number of target and OAR voxels, respectively; *D_pre_* and *D_max_* are the prescribed dose per fraction for the target and the maximum dose per fraction for the OAR, respectively, where *D_max_* is defined as a percentage of *D_pre_*; $D_{i}(\vec{N})$ is the actual physical or biological dose per fraction at voxel *i*; ∆*D_pre_* and ∆*D_max_* are normalization factors, and they are usually imposed equal to 0.025·*D_pre_* and 0.025·*D_max_*, respectively, where 0.025 is half of the estimated percental accuracy in dose calculation (5); *w*_T_ and *w_OAR_* are the weight factors associated with each VOI; finally


(5) $$\theta_{D_{\max}}=\theta (D_{i}(\vec{N})-D_{\max})=\{\begin{array}{ll} 1, & D_{i}(\vec{N})>D_{\max}\\ 0, & D_{i}(\vec{N})\leq D_{\max} \end{array}$$

is a Heaviside function in order to penalize only overdosage of OAR voxels, unlike the target, where both under- and overdosages are penalized.

#### 2.2.2 Dose Calculation

The interaction between a heavy ion beam and biological matter is very complex and leads to the creation of a mixed radiation field, due to the presence of ions with a very different linear energy transfer (LET) and the production of secondary particles caused by the fragmentation of the primary ions. The actual biological effect must be taken into account when calculating the dose in the case of a particle beam.

The physical dose (or absorbed dose) (5) at each voxel *i* is calculated as


(6) $$D_{i}^{phys}(\vec{N})={\vec{d}}_{i}^{T}\cdot \vec{N}=\sum_{j}d_{ij}N_{j}$$

where ${\vec{d}}_{i}^{T}$ is the transposed column vector of the dose correlation matrix, whose elements *d_ij_* represent the contribution from rasterpoint *j* to the dose at voxel *i* (8).

The biological dose (or RBE-weighted dose) in a voxel *i* is defined as the product between the physical dose $D_{i}^{phys}$ and the relative biological effectiveness *RBE_i_* (6):


(7) $$D_{i}^{bio}(\vec{N})=D_{i}^{phys}(\vec{N})\cdot RBE_{i}(\vec{N})$$

where the physical dose in each voxel *i* is the result of the superposition of several pencil beams (5), while *RBE* is a function of the tissue type and mixed radiation field, and it is calculated according to the local effect model (LEM) (6, 24–26).

Due to the stochastic nature of ion traversals and energy distributions, the biological damage for mixed radiation fields is estimated using Monte Carlo integration methods in the “classical” approach (6). Since this approach is very time-consuming, a faster method was developed, i.e., the so-called “low-dose” approximation (27), which allows to determine an analytical expression for the biological dose with an acceptable error of a few percent with respect to the “classical” approach in a therapeutic range of doses (6), i.e.,


(8) $$D_{bio}=\{\begin{array}{ll} \sqrt{\frac{-\ln\;S}{\beta_{X}}+{\left(\frac{\alpha_{X}}{2\beta_{X}}\right)}^{2}}-\left(\frac{\alpha_{X}}{2\beta_{X}}\right), & -\ln\;S\leq -\ln\;S_{t}\\ \frac{-\ln\;S+\ln\;S_{t}}{s_{\max}}+D_{t}, & -\ln\;S>-\ln\;S_{t} \end{array}$$

where the biological effect is


(9) $$-\ln\;S=\{\begin{array}{ll} (\bar{\beta }\;D_{phys}+\bar{\alpha })\;D_{phys}, & D_{phys}\leq D_{t}\\ (\bar{\beta }\;D_{t}+\bar{\alpha })\;D_{t}+(D_{phys}-D_{t})\;s_{\mathrm{max,}} & D_{phys}>D_{t} \end{array}$$

where *D_t_* and *S_max_* are respectively the dose threshold and the maximum slope at high doses used by the LEM, determining the transition from the linear-quadratic to a purely linear region of response, *S_t_* is the survival fraction at *D_t_*, *α_x_* and *β_x_* are the X-ray coefficients of the dose–response curve, and $\bar{\alpha }$ and $\bar{\beta }$ are the mixed field coefficients, derived by a Zaider–Rossi weighting (28) of the *α* and *β* parameters of each particle type and energy, composing the beam (27).

#### 2.2.3 Iterative Optimization Algorithms

The optimization problem consists in determining the optimal particle number for each spot *j*, i.e., the optimal dose distribution, *via* minimization of the cost function $\chi^{2}(\vec{N})$ . This requires to deal with a couple of problems, such as several ten thousands of rasterpoints to be handled as free parameters, a minimum number of particles for each rasterspot due to the technological limitations of the raster scanner, the presence of the Heaviside functions in the cost function, and the non-linearity of the biological dose. All this makes it impossible to solve the problem analytically and forces the use of fast and efficient algorithms. Several optimization methods exist, approaching the problem in different ways. In TRiP98, the type of algorithms already implemented belong to *line search methods*, which are commonly used and are based on the gradient of the cost function with respect to the particle numbers $\nabla \chi^{2}(\vec{N})$ .

The principle behind these iterative algorithms is as follows: at each iteration *k*, the vector of the particle numbers ${\vec{N}}_{k}$ is obtained such that the condition $\chi^{2}({\vec{N}}_{k+1})<\chi^{2}({\vec{N}}_{k})$ is true. The new particle numbers are calculated as


(10) $${\vec{N}}_{k+1}={\vec{N}}_{k}+\mu_{k}{\vec{h}}_{k}$$

With this parametrization, the multidimensional optimization problem is reduced to the determination of a minimization direction ${\vec{h}}_{k}$ and the estimation of a stepsize *μ_k_* along the search direction ${\vec{h}}_{k}$ . Repeating this calculation for a certain number of iterations, the actual vector of the particle numbers ${\vec{N}}_{k}$ converges towards the optimal vector ${\vec{N}}_{opt}$ . The starting values for particle numbers ${\vec{N}}_{0}$ are calculated during the preoptimization as described in Gemmel et al. (8).

Several iterative optimization algorithms are implemented in TRiP98. In this work, both the simplest one [steepest descent (SD)] (8), which consists in minimizing the cost function along its negative gradient, and the default one [Fletcher–Reeves variant of conjugated gradients (CGFR)] (9), which is faster because the minimization direction takes into account the previous successful iterations, have been employed. More details about iterative optimization algorithms used in this work and convergence tests are given in the **Supplementary Material (Section 1)**.

The optimal particle numbers can be obtained considering the two or more irradiation fields separately (single field optimization) or simultaneously (multiple field optimization) (8) during the optimization procedure. In particular, in this work, the second approach is used because it allows a better sparing of the critical biological structure, in particular for complex anatomy cases.

There are two methods to obtain the optimal particle numbers. The simplest approach consists in neglecting the variability of the biological effect, optimizing the absorbed dose, which depends in a linear way on the number of particles $\vec{N}$ , i.e., the actual dose in the cost function is $D_{i}(\vec{N})=D_{i}^{phys}(\vec{N})$ (physical optimization) (5). Instead, in the second approach, the actual dose is calculated according to equation 8, i.e., $D_{i}(\vec{N})=D_{i}^{bio}(\vec{N})$ , which depends in a non-linear way on the number of particles $\vec{N}$ (biological optimization) (6).

### 2.3 Implementation of Generalized Equivalent Uniform Dose-Based Optimization for Organs at Risk

The gEUD-based optimization allows to control the whole DVH shape of an OAR using a single objective, taking into account its volume effect in the optimization, by adjusting the prescribed value *gEUD_0_*, i.e., the desired dose level to be reached for each OAR, and the volume effect parameter *a*, which quantifies the volume effect of the OAR considered. In order to do this, an additional term for each OAR in the original cost function, with a quadratic penalty, is implemented in TRiP98, namely,


(11) $$\chi_{gEUD}^{2}(\vec{N})={\left(w_{OAR}\right)}^{2}\frac{{\left(gEUD_{0}-gEUD(\vec{N})\right)}^{2}}{{\left(\Delta gEUD_{0}\right)}^{2}}\theta_{gEUD}$$

where


(12) $$gEUD(\vec{N})={\left(\frac{1}{M_{OAR}}\sum_{i=1}^{M_{OAR}}{\left(D_{i}(\vec{N})\right)}^{a}\right)}^{\frac{1}{a}}$$

is the actual value, *M_OAR_* is the total number of voxels for a single OAR, ∆*gEUD_0_* = 0.025 *gEUD_0_* is the normalization factor, *w_OAR_* is the weight factor, and


(13) $$\theta_{gEUD}=\theta (gEUD(\vec{N})-gEUD_{0})=\{\begin{array}{ll} 1, & gEUD(\vec{N})>gEUD_{0}\\ 0, & gEUD(\vec{N})\leq gEUD_{0} \end{array}$$

is a Heaviside function in order to penalize OAR with actual gEUD larger than the prescribed value *gEUD_0_*.

Therefore, the total cost function is


(14) $$\chi^{2}(\vec{N})=\chi_{D_{pre}/D_{\max}}^{2}(\vec{N})+\chi_{gEUD}^{2}(\vec{N})$$

and it is possible to decide whether to optimize the dose distribution for a given organ by imposing a maximum dose or a prescribed gEUD and also to choose different values of *D_max_* or *gEUD_0_* for each OAR considered.

In principle, by decreasing *gEUD_0_*, one can achieve a lower *gEUD* for a given organ, i.e., a larger sparing. The expected result of changes in *a* is to change the dose range where the organ sparing will be maximized: for example, selecting *a = 1* the whole area under the DVH curve should be minimized, while for *a >>* 1, the best DVH will be obtained in terms of sparing at high doses.

In the following paragraphs, the solutions in the case of physical and biological optimization are presented.

#### 2.3.1 Solution for Physical Optimization

The fundamental step to solve the optimization problem is the determination of the gradient of the cost function $\chi^{2}(\vec{N})$ with respect to the particle numbers $\vec{N}$ . In the case of physical optimization, i.e., neglecting *RBE* in dose calculation, the gradient of the total cost function is calculated as


(15) $$\nabla \chi^{2}(\vec{N})=\nabla \chi_{D_{pre/}D_{\max}}^{2}(\vec{N})+\nabla \chi_{gEUD}^{2}(\vec{N})$$

where the voxel-dose-based term is


(16) $$\begin{array}{c} \nabla \chi_{D_{pre}/D_{\max}}^{2}=-2(w_{T}{)}^{2}\sum_{i=1}^{M_{T}}\frac{\left(D_{pre}-D_{i}^{phys}\right)}{{\left(\Delta D_{pre}\right)}^{2}}\cdot \nabla D_{i}^{phys}\\ -2(w_{OAR}{)}^{2}\sum_{i=1}^{M_{OAR}}\frac{\left(D_{\max}-D_{i}^{phys}\right)}{{\left(\Delta D_{\max}\right)}^{2}}\cdot \nabla D_{i}^{phys}\cdot \theta_{D_{\max}} \end{array}$$

and the new gEUD-based term is


(17) $$\begin{array}{c} \nabla \chi_{gEUD}^{2}=-2(w_{OAR}{)}^{2}\frac{\left(gEUD_{0}-gEUD\right)}{{\left(\Delta gEUD_{0}\right)}^{2}}{\left(\frac{1}{M_{OAR}}\right)}^{\frac{1}{\alpha }}\\ \times {\left(\sum_{i=1}^{M_{OAR}}{\left(D_{i}^{phys}\right)}^{a}\right)}^{\frac{1}{\alpha }-1}\sum_{i=1}^{M_{OAR}}{\left(D_{i}^{phys}\right)}^{a-1}\cdot \nabla D_{i}^{phys}\cdot \theta_{gEUD} \end{array}$$

Thanks to the chain rule in the derivation, the task becomes the calculation of the absorbed dose gradient for each voxel *i* from equation 6, which is calculated as $\nabla D_{i}^{phys}={\vec{d}}_{i}^{T}$ , where ${\vec{d}}_{i}^{T}$ is the transposed column vector of the dose correlation matrix already introduced.

The second important step is the determination of a scalar *μ_k_*, i.e., the stepsize, for each iteration *k*, solving the following equation:


(18) $$\frac{d\chi^{2}({\vec{N}}_{k}+\mu_{k}{\vec{h}}_{k})}{d\mu_{k}}=\frac{d\chi_{D_{pre}/D_{\max}}^{2}({\vec{N}}_{k}+\mu_{k}{\vec{h}}_{k})}{d\mu_{k}}+\frac{d\chi_{gEUD}^{2}({\vec{N}}_{k}+\mu_{k}{\vec{h}}_{k})}{d\mu_{k}}=0$$

for *μ_k_* and for each iteration *k*, where the physical dose at iteration *k* + 1 is calculated as $D_{i}^{phys}({\vec{N}}_{k+1})={\vec{d}}_{i}^{T}{\vec{N}}_{k+1}={\vec{d}}_{i}^{T}({\vec{N}}_{k}+\mu_{k}{\vec{h}}_{k})$ .

In the case of physical optimization and for OARs with large volume effects, namely, considering *a* = 1, equation 18 is solved analytically because all terms are linear in *μ_k_*. Instead, in the case of *a* > 1 due to the non-linearity of the gEUD-based term, we made two approximations: linearization of $gEUD(D_{i}^{phys}({\vec{N}}_{k+1}))$ for small dose variation in each voxel *i*, i.e., $\mu_{k}{\vec{d}}_{i}^{T}{\vec{h}}_{k}\ll {\vec{d}}_{i}^{T}{\vec{N}}_{k}$ , and large volume effect approximation, i.e., considering *a* = 1. The expression obtained for the stepsize $\mu_{k}^{phys}$ , in this case, is reported in the **Supplementary Material (Section 2)**.

#### 2.3.2 Solution for Biological Optimization

Biological effectiveness and its relative variation for a high Z particle like carbon are not negligible. For this reason, we focused on biological optimization, which consists in considering the total cost function defined in equation 14, but where the actual dose *D_i_* ( $\vec{N}$ ) at each voxel *i* is calculated as the RBE-weighted dose $D_{i}^{bio}$ , i.e., according to equation 7. The difficulty is that this expression is highly non-linear because both the absorbed dose $D_{i}^{phys}$ and the *RBE_i_* depend on the vector of particle numbers $\vec{N}$ . An approach to solve the problem is now presented. As for the physical case, also for biological optimization, it is necessary to calculate the gradient of the total cost function and estimate the stepsize.

The expression of the gradient of the total cost function is the same as for the physical optimization case thanks to the chain rule in the derivation; the only difference is the presence of the biological dose gradient with respect to the number of particles:


(19) $$\nabla D_{i}^{bio}=RBE_{i}\cdot \nabla D_{i}^{phys}+D_{i}^{phys}\cdot \nabla RBE_{i}$$

where the first term is the physical gradient component, while the second term is the biological gradient component.

There are several ways to calculate this gradient already implemented in TRiP98: the simplest approach is the classical method (6), in which ∇*RBE_i_* is neglected, namely, the RBE is considered as a constant. However, in this way, the minimization direction is not optimally determined, and we may have accuracy problems during optimization. Therefore, the approach used in this work is based on the so-called “low-dose” approximation for a mixed radiation field (27), which allows to obtain an analytical expression for the biological dose and its gradient, according to equation 8, in a fast way.

The second element necessary to solve the optimization problem is the determination of a stepsize $\mu_{k}^{bio}$ for each iteration *k*, in principle solving equation 18. But due to the non-linearity of the biological dose with $\vec{N}$ , it is not possible to obtain an analytical expression for the stepsize *μ_k_*. For this reason, only an estimate of the true solution can be obtained, which approximately fulfills the equation.

The most common method used in TRiP98 is based on the calculation of the stepsize $\mu_{k}^{phys}$ solving equation 18; then, using “damping factor” *f*, an estimate of $\mu_{k}^{bio}$ for each iteration *k* is thus obtained as


(20) $$\mu_{bio}=f\cdot \mu_{phys}$$

Testing different “damping factor” values, it is noticed that a reasonable value is *f* = 0.5, as it was reported in Gemmel et al. (8).

### 2.4 Patient and Plan Parameters

In order to study the role of the cost function parameters *gEUD_0_* and *a*, the gEUD-based optimization is tested considering a plan containing the proximal parotid gland as an OAR, in addition to the tumor (chordoma). Also the brainstem is considered, as an additional OAR, in order to have a clinically realistic plan. The tumor is irradiated using two nearly opposite fields, with (couch) angles −100° and 75°, according to the original plan. The uniform prescribed dose *D_pre_* for the target is 3 Gy, according to the original prescription for a single fraction of the patient case, while the parotid is optimized with different combinations of *gEUD_0_* and *a* values. The SD algorithm was used.

The new gEUD-based optimization approach is tested for different sample plans of patients treated for head and neck cancers during the GSI pilot project. The tumor is a chordoma located in the skull base, while the typical OARs in this region are organs with a small volume effect, like the spinal cord, the brainstem, the optic nerves, and the chiasm, for which the most important dose level is the maximum dose. But there are also important glands located in correspondence of the cheeks with a large volume effect, i.e., the parotid glands, for which the aim is to reduce the mean dose, in order to reduce the probability of complications (reduction of the salivary flow, speech and taste alterations, etc.). For all these plans, a multiple field optimization of the biological dose is performed, using the CGFR algorithm. Moreover, the plans obtained using the gEUD-based optimization are compared with the results coming from the standard voxel-dose-based approach. The prescriptions for the plans for both voxel-dose-based and gEUD-based optimization are reported in **Table 1**.

**Table 1:** Cost function parameters for plans 135 and 335.

	Plan 135	
**VOI**	**Voxel-dose-based opt.**	**gEUD-based opt.**
Target	*D_pre_* = 3.00 Gy, *w_T_* = 1	*D_pre_* = 3.00 Gy, *w_T_* = 1
Spinal cord	*D_max_* = 1.50 Gy, *w_OAR_* = 1	*gEUD*_0_ = 1.30 Gy, *a* = 20, *w_OAR_* = 25
	**Plan 335**	
**VOI**	**Voxel-dose-based opt.**	**gEUD-based opt.**
Target	*D_pre_* = 3.00 Gy, *w_T_* = 1	*D_pre_* = 3.00 Gy, *w_T_* = 1
Right parotid	*D_max_* = 2.25 Gy, *w_OAR_* = 1	*gEUD*_0_ = 0.50 Gy, *a* = 1, *w_OAR_* = 20
Brainstem	*D_max_* = 2.25 Gy, *w_OAR_* = 1	*gEUD*_0_ = 1.90 Gy, *a* = 20, *w_OAR_* = 20
Spinal cord	*D_max_* = 1.80 Gy, *w_OAR_* = 1	*gEUD*_0_ = 1.40 Gy, *a* = 20, *w_OAR_* = 20
Right optic nerve	*D_max_* = 1.50 Gy, *w_OAR_* = 1	*gEUD*_0_ = 0.20 Gy, *a* = 20, *w_OAR_* = 20
Left optic nerve	*D_max_* = 1.50 Gy, *w_OAR_* = 1	*gEUD*_0_ = 1.30 Gy, *a* = 20, *w_OAR_* = 20
Chiasm	*D_max_* = 1.50 Gy, *w_OAR_* = 1	*gEUD*_0_ = 1.10 Gy, *a* = 20, *w_OAR_* = 20

VOI, volume of interest; gEUD, generalized equivalent uniform dose.

A typical situation of chordoma case is patient number 135 from the patient database of the GSI pilot project. This represents a very complex anatomical geometry, where the tumor is wrapped around the spinal cord, which is the OAR considered in this plan. This tumor is treated using two nearly opposite fields, with (couch) angles −100° and 104°, according to the original plan.

Another typical treatment plan is patient 335, which contains multiple OARs with small volume effects, like the spinal cord, the brainstem, the right and left optic nerves, the chiasm, but also the right parotid gland (proximal in this irradiation geometry), with a large volume effect. The tumor is irradiated using two nearly opposite fields, with (couch) angles −100° and 75°, according to the original plan.

A further investigation is done with this treatment plan. In fact, in the original plan for patient 335, only the proximal (right) parotid gland was considered. Therefore, the idea is to consider also the distal (left) parotid gland in order to see what happens if it is optimized using the gEUD-based approach. The aim is to reduce the mean dose received by both parotids. For this reason, an additional objective is considered for the left parotid in the definition of the total cost function; in particular, a volume effect parameter equal to 1 and a *gEUD_0_* equal to 0.60 Gy are used for both glands. The objectives for the other organs are the same as in the previous plan (see **Table 1**).

Additional results about patient plans optimization are reported in the **Supplementary Material (Section 3)**.

The estimates of NTCP presented in (29) for the parotids correspond to a dose per fraction of 2 Gy, while the dose per fraction prescribed in the optimization of our work is 3 Gy. This choice is due to the fact that the treatment plans considered in this work come from the GSI pilot project (which is a standard reference for TRiP98 implementations), and for this reason, we decided not to change the prescribed dose values. However, this deviation would only involve at most an underestimation of the improvement obtained in terms of NTCP with the new approach based on gEUD compared to the method based on the maximum dose. A possible way to solve this limitation is to calculate the mean dose to the parotids in terms of EQD2 and then to estimate their NTCP, i.e.,


(21) $$EQD2=D_{tot}\left(\frac{\alpha /\beta +D_{tot}/N_{frac}}{\alpha /\beta +2\;Gy}\right)$$

where *D_tot_* is the total dose, *N_frac_* is the number of fractions, and *α/β* = 2 Gy in this case.

## 3 Results

### 3.1 Role of the Cost Function Parameters

This section reports the results obtained for the study of the cost function parameters during the optimization, in particular *gEUD_0_* and *a* for an OAR. The patient considered has been described in Section 2.4.

**Figure 2:**
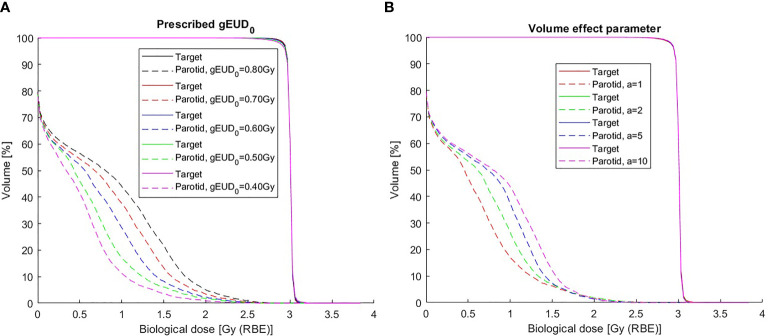
Dose–volume histograms for a chordoma patient (same as in **Figure 3**). **(A)** For different *gEUD_0_* to the parotid. Cost function parameters: *D_pre_* = 3.00 Gy, *w_T_ = 1* (target); w*_OAR_ = 20*, *gEUD*_0_ = 0.80, 0.70, 0.60, 0.50, 0.40 Gy, and *a*= 1 (parotid). **(B)** For different *a* values of the parotid. Cost function parameters: *D_pre_* = 3.00 Gy, *w_T_ = 1* (target); w*_OAR_ = 20*, (*gEUD*_0_, *a*) = [(0.50 Gy, 1), (0.80 Gy, 2), (1.15 Gy, 5), (1.45 Gy, 10)] (parotid).

**Figure 3:**
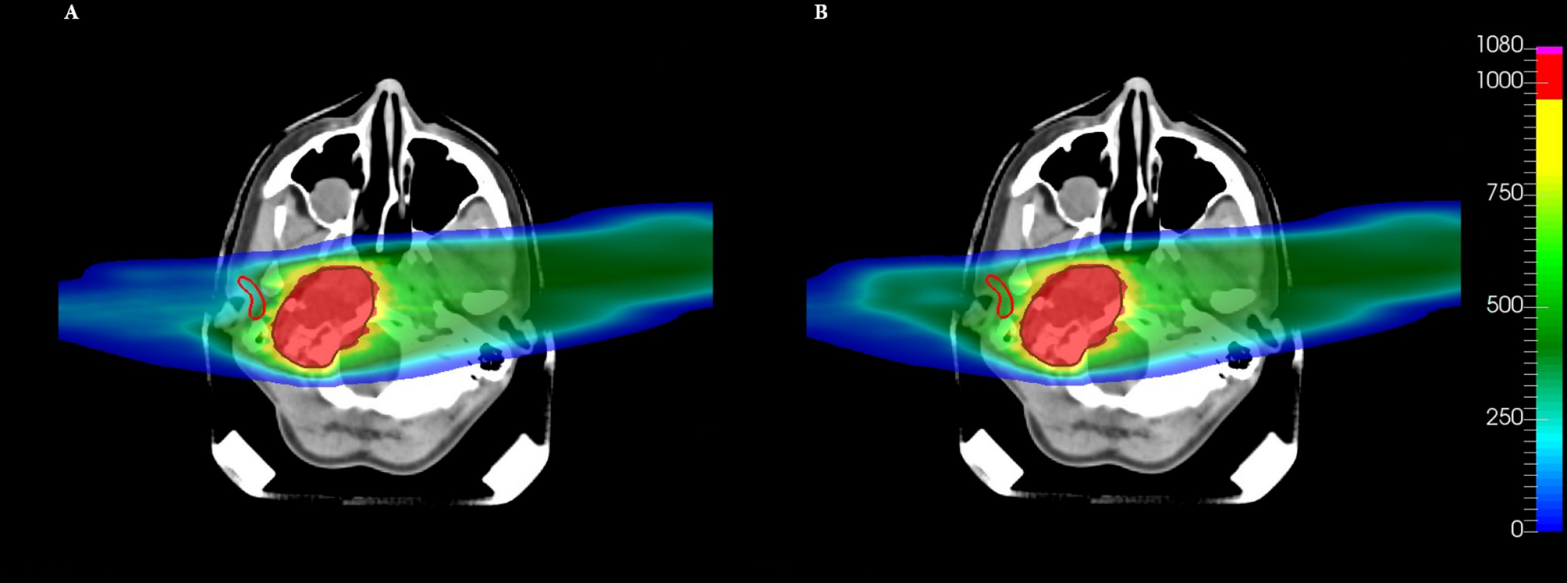
Comparison of dose distributions on a CT slice for different volume effect parameters *a* of the parotid. Cost function parameters: **(A)** *gEUD*_0_ = 0.50 Gy, *a* = 1 (red DVH in **Figure 2B**). **(B)** *gEUD*_0_ = 1.45 Gy, *a* = 10 (violet DVH in **Figure 2B**). The target (brown contour) and the right parotid (red contour) are shown. The dose levels are plotted in per mil of the prescribed dose.

The DVH in **Figure 2A** shows the dependence of the dose distributions for values of *gEUD_0_* between 0.80 and 0.40 Gy, for a fixed weight factor *w_OAR_* = 20 and volume effect parameter *a* = 1. As expected, decreasing *gEUD_0_*, the mean dose *D_mean_* decreases from 0.81 to 0.44 Gy. Instead, the different impact on the DVH shape is visible in **Figure 2B**, using different values for the volume effect parameter *a*, with *a* = 1 minimizing the mean dose, while as *a* increases, this reduction shifts to regions of the DVH that receive higher doses (the maximum dose *D_max_* decreases with increasing *a*, in particular from 2.69 Gy for *a* = 1 to 2.38 Gy for *a* = 10). It should be noted that in **Figure 2B** the *gEUD_0_* value is changed correspondingly to the variation of the *a* value to reflect the different regions of the optimization, by obtaining *D_max_* values almost constant. All these parotid dose distributions are achievable without affecting the target dose. This effect is also visible in the 2D dose distributions (**Figure 3**).

Therefore, by appropriately choosing a pair of values for the volume effect parameter and for the prescribed gEUD, it is possible to finely control the shape of the DVH, depending on the type of biological architecture of the organ under consideration. Of course, the levels of control and variability of the DVH shape depend not on the gEUD parameters only but also on how the different components of the whole cost function interact with one another.

### 3.2 Comparison Between Voxel-Dose-Based and Generalized Equivalent Uniform Dose-Based Optimization

This section shows the results obtained for patient plans optimized with the gEUD-based and voxel-dose-based approach. The plan parameters have been described in Section 2.4.

**Figure 4:**
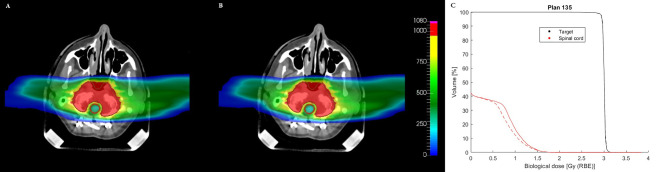
Comparison of dose distributions on a CT slice for patient plan 135, obtained with **(A)** voxel-dose-based and **(B)** gEUD-based optimization. The target (brown contour) and the spinal cord (red contour) are shown. The dose levels are plotted in per mil of the prescribed dose. **(C)** Comparison of DVHs obtained with voxel-dose-based (solid line) and gEUD-based (dashed line) optimization for patient plan 135. gEUD, generalized equivalent uniform dose; DVH, dose–volume histogram.

In the case of patient number 135, from the gEUD values and the maximum doses obtained (**Table 2**) and from the DVHs (**Figure 4C**), it is evident that by using a volume effect parameter equal to 20, it is possible to obtain very similar plans using two different optimization approaches: in particular the *gEUD* values of the spinal cord are equivalent, and the target DVHs are identical. This is also confirmed by the dose distributions as shown in the CT slices in **Figures 4A, B**. The choice of *a* = 20 is due to the fact that the spinal cord is a typical serial organ and therefore requires a large volume effect parameter value. Furthermore, considering, for example, *a* = 15 or *a* = 25 in the optimization, the maximum dose is stable (*D_max_* = 1.83 Gy and *D_max_* = 1.79 Gy, respectively), and for this reason, the value *a* = 20 was chosen.

**Table 2:** gEUD and other dosimetric indexes for the VOIs of plan 135.

Parameter	Voxel-dose-based opt.	gEUD-based opt.
	**Target**	
*D_min_*	2.20 Gy	2.17 Gy
*D_max_*	3.24 Gy	3.24 Gy
*D_mean_*	3.00 Gy	3.00 Gy
*CI*	1.25	1.25
	**Spinal cord**	
*gEUD* (*a* = 20)	1.37 Gy	1.35 Gy
*D_max_*	1.92 Gy	1.83 Gy

CI = volume 95% isodose / volume VOI.

gEUD, generalized equivalent uniform dose; OAR, organ at risk; VOI, volume of interest.

**Figure 5:**
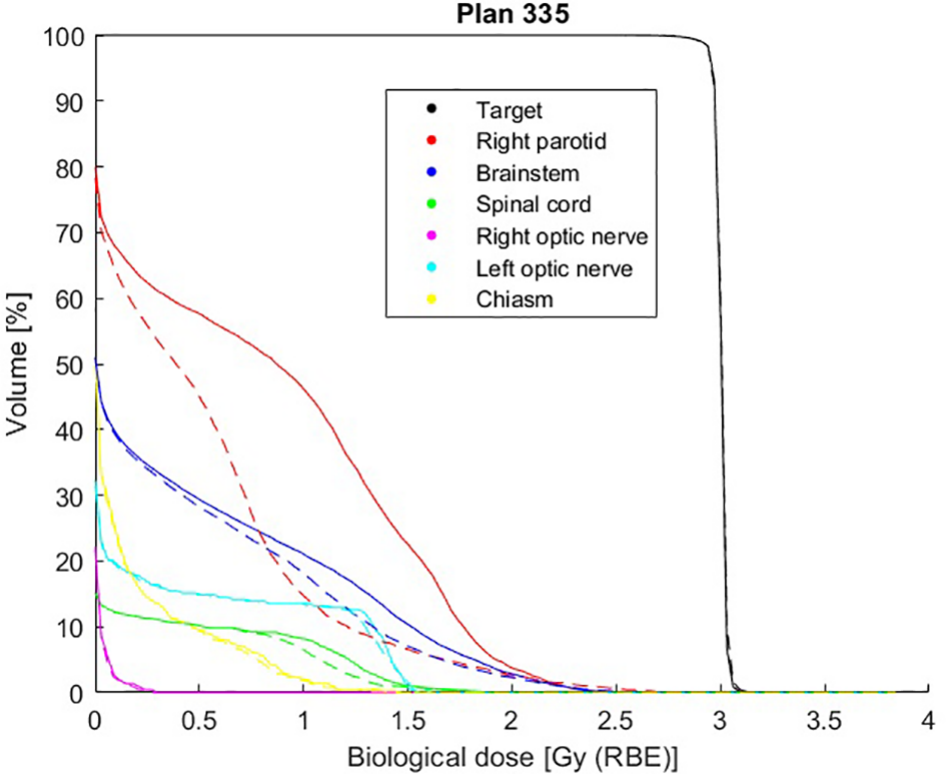
Comparison of dose–volume histograms (DVHs) obtained with voxel-dose-based (solid line) and generalized equivalent uniform dose (gEUD)-based (dashed line) optimization for patient plan 335.

**Figure 6:**
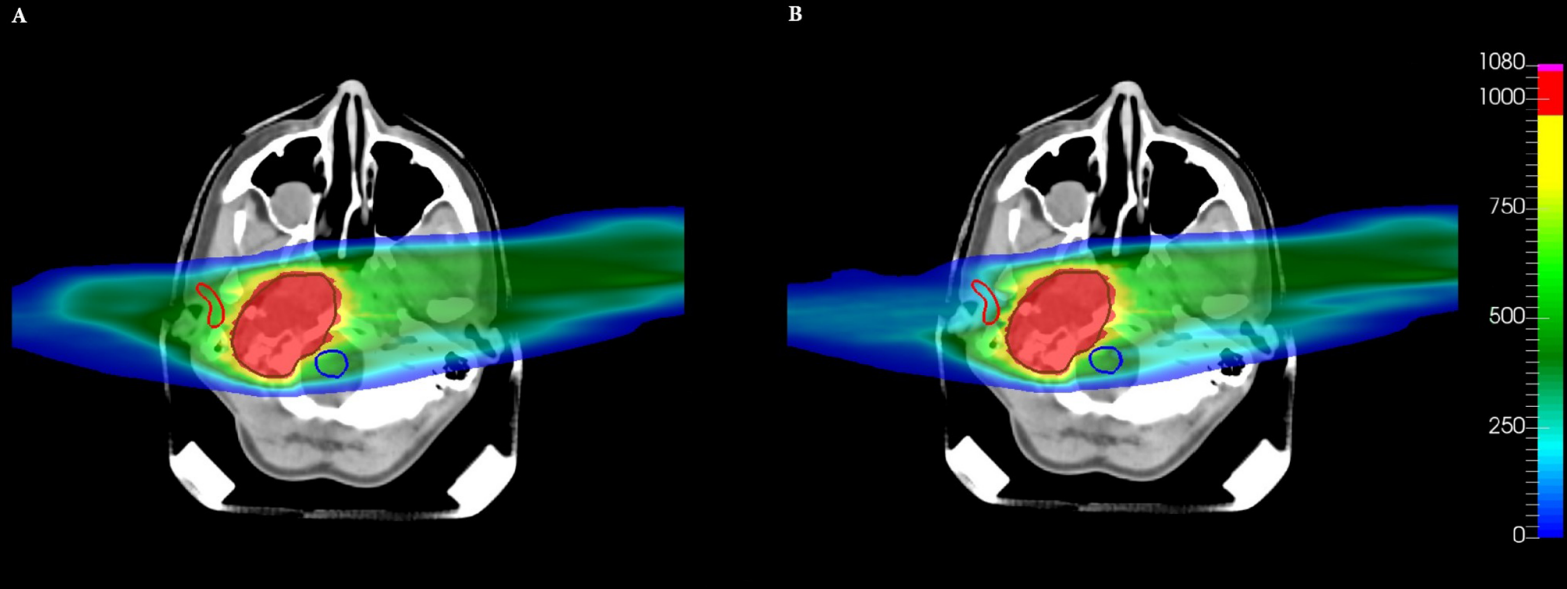
Comparison of dose distributions on a CT slice for patient plan 335, obtained with **(A)** voxel-dose-based and **(B)** generalized equivalent uniform dose (gEUD)-based optimization. The target (brown contour), the right parotid (red contour), and the brainstem (blue contour) are shown. The dose levels are plotted in per mil of the prescribed dose.

For patient number 335, observing the gEUD values and the maximum doses of the OARs coming from the optimization (**Table 3**), the DVHs (**Figure 5**), and the dose distributions (**Figure 6**), it is possible to conclude that the optimization of a complex plan, containing many biological structures with a small volume effect, using gEUD-based objectives is feasible; in particular, the DVHs of organs with a small volume effect are equivalent from a clinical point of view, but also the gEUD values for these organs, with *a* = 20, are the same.

**Table 3:** gEUD, NTCP and other indexes for the VOIs of plan 335.

Parameter	Voxel-dose-based opt.	gEUD-based opt.
	**Target**	
*D_min_*	2.52 Gy	2.16 Gy
*D_max_*	3.17 Gy	3.39 Gy
*D_mean_*	3.00 Gy	3.00 Gy
*CI*	1.24	1.27
	**Right parotid**	
*gEUD* (*a* = 1)	0.82 Gy	0.50 Gy
*NTCP*	11.09%	4.37%
*D_max_*	2.51 Gy	2.80 Gy
	**Brainstem**	
*gEUD* (*a* = 20)	1.89 Gy	1.93 Gy
*D_max_*	2.53 Gy	2.62 Gy
	**Spinal cord**	
*gEUD* (*a* = 20)	1.40 Gy	1.40 Gy
*D_max_*	1.89 Gy	1.93 Gy
	**Right optic nerve**	
*gEUD* (*a* = 20)	0.22 Gy	0.19 Gy
*D_max_*	0.29 Gy	0.25 Gy
	**Left optic nerve**	
*gEUD* (*a* = 20)	1.29 Gy	1.30 Gy
*D_max_*	1.53 Gy	1.60 Gy
	**Chiasm**	
*gEUD* (*a* = 20)	1.08 Gy	1.04 Gy
*D_max_*	1.44 Gy	1.39 Gy

CI = volume 95% isodose / volume VOI.

gEUD, generalized equivalent uniform dose; OAR, organ at risk; NTCP, normal tissue complication probability; VOI, volume of interest.

In addition to that, the most important result obtained here is that the gEUD-based optimization allows to reduce the mean dose received by the proximal parotid gland, considering a volume effect parameter equal to 1 (see **Table 3**), without losing target coverage; it is also visible by observing the dose distributions in **Figure 6**. This is very important because the probability of a complication for this biological structure, i.e., the NTCP, is linked with the mean dose. Therefore, a gEUD reduction corresponds to a NTCP reduction. This is quantified using the LKB model for NTCP, according to equations 2 and 3, and considering the parameters proposed by Dijkema et al. (29), where *n = a* = 1 was fixed in the fit, and the values of *TD_50_* and *m* and their 95% CIs were *TD*_50_ = 39.9 Gy (37.3–42.8) and *m* = 0.40 (0.34–0.51).

**Figure 7:**
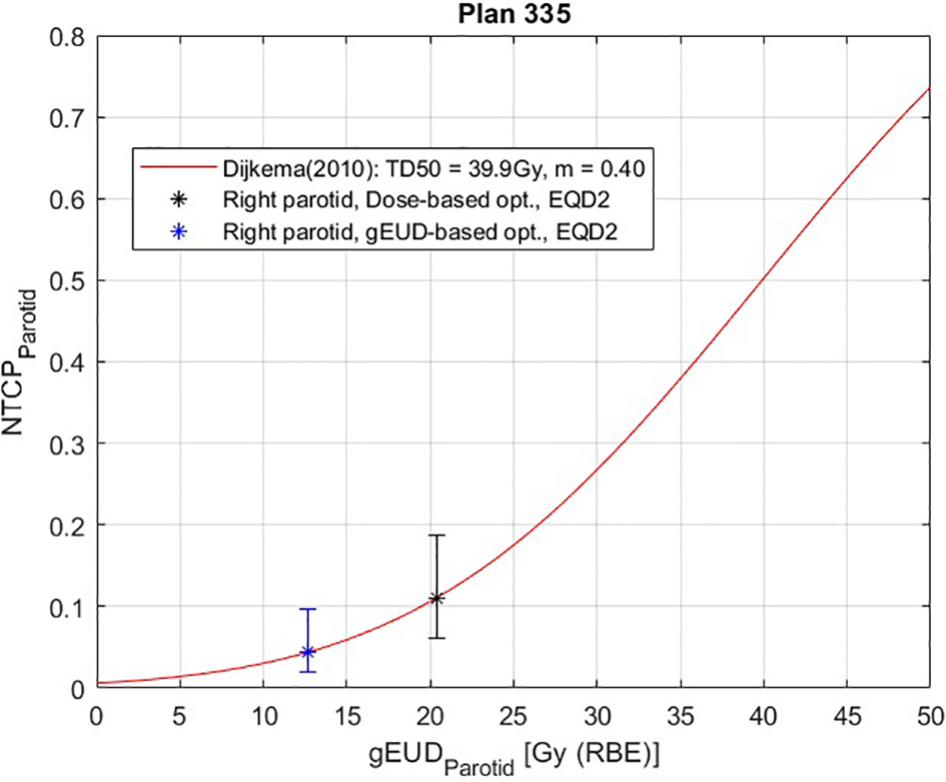
Normal tissue complication probability (NTCP) curve for the right parotid gland of patient plan 335, calculated according to Lyman–Kutcher–Burman (LKB model) using the parameters obtained by Dijkema et al. (29). The error bars were calculated considering the maximum and minimum NTCP values coming from the combination of the extreme values of the parameters *TD*_50_ and *m* (95%CIs): *TD*_50_ = 37.3 Gy and *m *= 0.51 for the highest NTCP value, and *TD*_50_ = 42.8 Gy and *m *= 0.34 for the lowest NTCP. A therapeutic plan of 20 fractions of 3 Gy is considered, with EQD2 calculation.

For this purpose, a complete treatment plan of 20 fractions of 3 Gy is considered. Then the NTCP curve for the right parotid is plotted as a function of the *gEUD* for *a* = 1, i.e., *D_mean_*; furthermore, the NTCP values corresponding to the gEUD values obtained with the two optimization methods are calculated, and they are plotted in **Figure 7**. In particular, for this plan, the NTCP of the proximal parotid is reduced from 6.98% to 3.09%, i.e., by a factor of 2.3, using the gEUD-based optimization. This means a higher sparing of the parotid gland using this new optimization approach. Considering EQD2 calculation according to equation 21, the NTCP is reduced from 11.09% to 4.37%, i.e., by a factor of 2.5.

**Figure 8:**
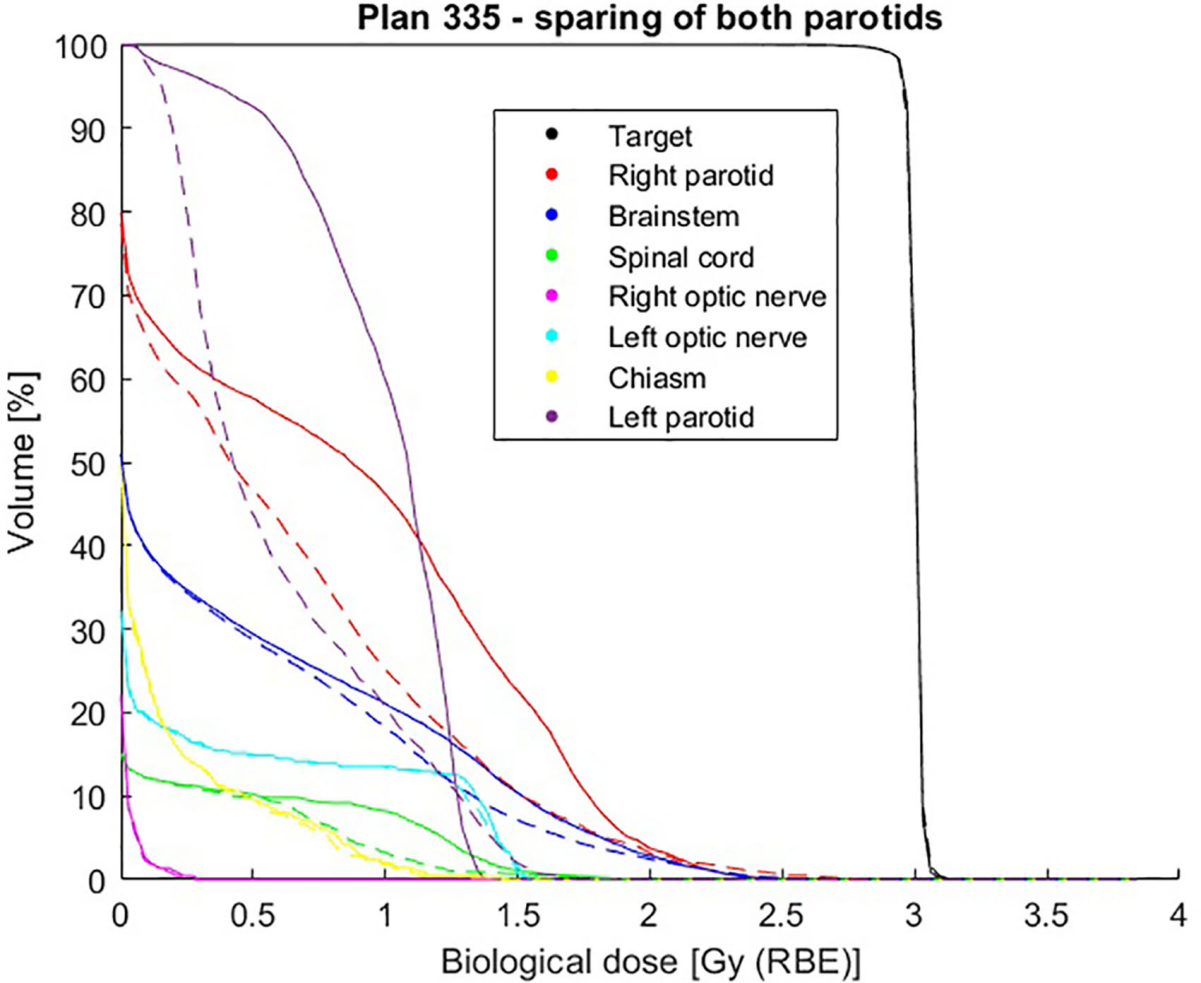
Comparison of dose–volume histograms (DVHs) obtained with voxel-dose-based (solid line) and generalized equivalent uniform dose (gEUD)-based (dashed line) optimization for patient plan 335, considering both parotid glands.

**Figure 9:**
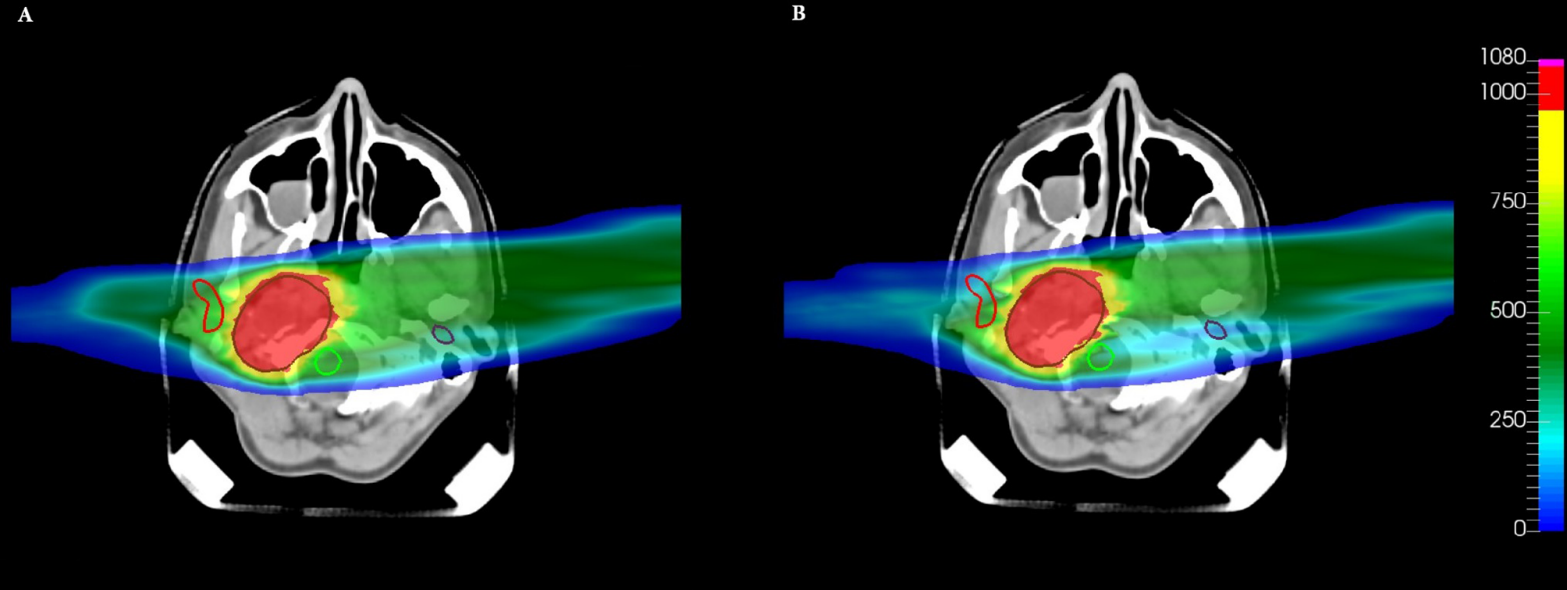
Comparison of dose distributions on a CT slice for patient plan 335 considering both parotid glands, obtained with **(A)** voxel-dose-based and **(B)** generalized equivalent uniform dose (gEUD)-based optimization. The target (brown contour), the right parotid (red contour), the left parotid (violet contour), and the spinal cord (green contour) are shown. The dose levels are plotted in per mil of the prescribed dose.

Considering both parotid glands for patient number 335, as can be seen from **Table 4** and from **Figures 8**, **9**, a mean dose reduction for both parotids is achieved using gEUD-based optimization instead of imposing a maximum dose for each gland. Note that a further reduction of the prescribed *D_max_*, besides, it does not correlate directly with a mean dose reduction, leads to a worsening of the target coverage, and we chose to compare plans with the same level of target coverage.

**Table 4:** gEUD, NTCP and other indexes for the VOIs of plan 335, considering both parotid glands.

Parameter	Voxel-dose-based opt.	gEUD-based opt.
	**Target**	
*D_min_*	2.52 Gy	2.42 Gy
*D_max_*	3.17 Gy	3.20 Gy
*D_mean_*	3.00 Gy	3.00 Gy
*CI*	1.24	1.24
	**Right parotid**	
*gEUD* (*a* = 1)	0.82 Gy	0.60 Gy
*NTCP*	11.10%	5.94%
*D_max_*	2.51 Gy	2.82 Gy
	**Left parotid**	
*gEUD* (*a* = 1)	0.98 Gy	0.59 Gy
*NTCP*	16.92%	5.78%
*D_max_*	1.37 Gy	1.70 Gy
	**Brainstem**	
*gEUD* (*a* = 20)	1.89 Gy	1.94 Gy
*D_max_*	2.53 Gy	2.62 Gy
	**Spinal cord**	
*gEUD* (*a* = 20)	1.40 Gy	1.40 Gy
*D_max_*	1.89 Gy	1.95 Gy
	**Right optic nerve**	
*gEUD* (*a* = 20)	0.22 Gy	0.19 Gy
*D_max_*	0.29 Gy	0.25 Gy
	**Left optic nerve**	
*gEUD* (*a* = 20)	1.29 Gy	1.30 Gy
*D_max_*	1.54 Gy	1.58 Gy
	**Chiasm**	
*gEUD* (*a* = 20)	1.08 Gy	1.04 Gy
*D_max_*	1.44 Gy	1.39 Gy

CI = volume 95% isodose / volume VOI.

gEUD, generalized equivalent uniform dose; OAR, organ at risk; NTCP, normal tissue complication probability; VOI, volume of interest.

**Figure 10:**
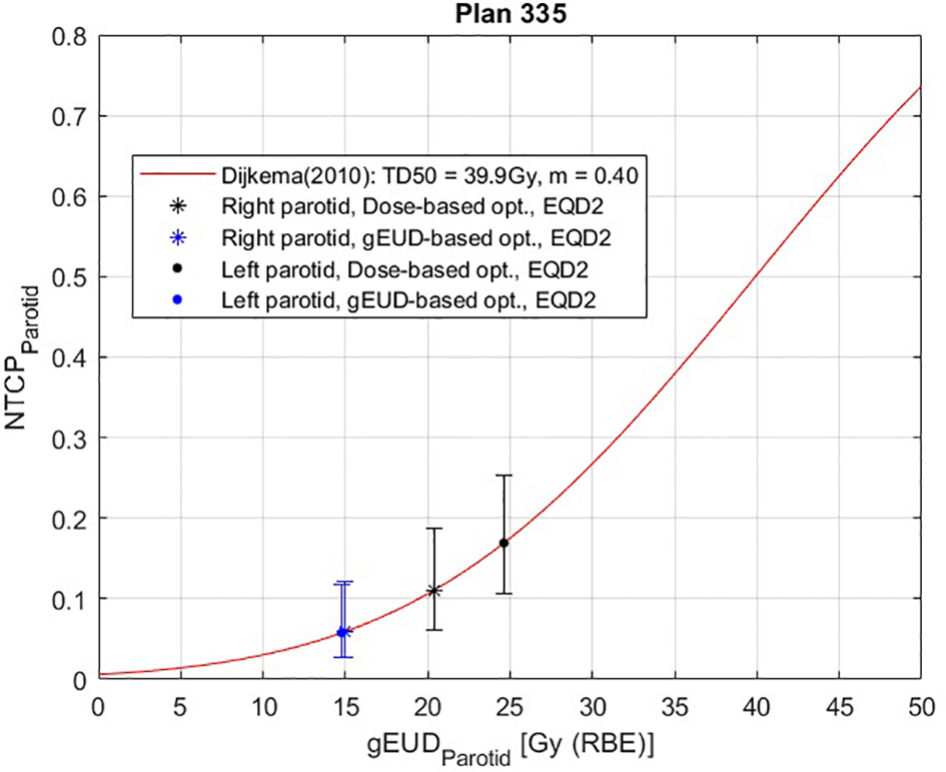
Normal tissue complication probability (NTCP) curve for the parotid glands of patient plan 335, calculated according to Lyman–Kutcher–Burman (LKB) model, using the parameters obtained by Dijkema et al. (29). The error bars were calculated as before. A therapeutic plan of 20 fractions of 3 Gy is considered, with EQD2 calculation.

Furthermore, from the NTCP curve in **Figure 10**, a reduction of NTCP for both parotids can be seen, in particular from 6.98% to 4.03% for the proximal one and from 10.28% to 3.93% for the distal one, while considering EQD2 calculation according to equation 21, the NTCP is reduced from 11.10% to 4.37% (proximal parotid) and from 16.92% to 5.78% (distal parotid).

## 4 Discussion

In this work, a possible approach of the gEUD-based optimization is implemented for the first time in TRiP98 as an alternative to the standard voxel-dose-based criteria. The resulting optimization method is able to account for RBE weighting of the dose and volume effects at the same time, i.e., a double level of biologically driven treatment planning.

From studying the cost function parameters during the optimization procedure, it emerges that it is possible to obtain different dose distributions for a given OAR using various combinations of prescription *gEUD_0_* and volume effect parameter *a*. In particular, as *gEUD_0_* decreases, it allows a greater sparing of the OAR considered, while as the parameter *a* increases, the DVH of the OAR takes very different shapes. For example, for *a =* 1, there is a decrease in the volume receiving doses close to the mean dose, while for a >> 1, there is a decrease in the volume receiving higher doses, as expected, thus showing high flexibility in planning criteria. This result is very important since gEUD is closely linked to the concept of NTCP, and therefore a decrease of gEUD leads to a reduction of NTCP. This is exactly what happens for patient plan 335 (**Figures 5**, **6**, **8**, **9**), where a reduction of *gEUD* for *a =* 1 in the case of the parotid involves a greater sparing of this gland and a reduction of the risk of complications quantified in terms of NTCP (**Figures 7**, **10**). Even more importantly, this occurs both considering the single parotid or both glands during the optimization and also without losing control of the target DVH. This means that, in principle, by choosing a reasonable combination of *gEUD_0_* and *a*, it is possible to reduce the probability of a complication for a given OAR by imposing a single objective during the optimization, formalizing it by a quadratic term during the definition of the cost function. Obviously, the effect of greater sparing of healthy organs will be more evident for organs with a large volume effect, as in the case of the parotid gland, compared to purely serial organs, in which the probability of complications is linked to the maximum dose, as for the spinal cord of patient plan 135 (**Figure 4**), where similar results are obtained using voxel-dose-based or gEUD-based optimization. But at the same time, this result can be seen as the possibility of using gEUD-based optimization for any type of organ, achieving improvements in the case of organs with large volume effect or similar results for organs with small volume effect with respect to the standard criteria, as in the present case, based on a maximum dose as an objective.

A possible limitation of this approach is that for many organs, there are no precise estimates for the volume effect parameter *a*, but only reasonable values from clinical studies. There is also a lack of knowledge of the specific tolerances for each organ in terms of gEUD. Therefore, it is necessary to test different combinations of *gEUD_0_* and *a* in order to identify the couple that leads to satisfactory results in terms of dose distributions and estimates of NTCP. On the other hand, a similar limitation is shared by the maximum dose criteria since such values are also associated with uncertainties.

It should be also noted that the large improvement observed in **Figures 5**, **8** would be probably reduced when compared to a voxel-dose-based objective including several points. We decided to directly implement the gEUD-based optimization instead of the possibility to add several DVH point constraints considering also the arbitrarity of such points selection.

As described in Section 2.2.3, the optimization task in TRiP98 is based on iterative algorithms that belong to *exact line search methods*, which require the calculation of a minimization direction and a stepsize in an analytical way. This approach, due to the non-linearity of the problem, imposed the use of some specific approximations: the linearization of the gEUD-based objective (in Section 2.3.1) and the use of a “damping factor” (in Section 2.3.2) in order to obtain an analytical expression of the stepsize for biological optimization. A possible simplification of this approach could be, as an alternative, the implementation of a numerical approach, like the *backtracking line search method* [e.g., (30)]. The latter is a more general method to get an approximated value of the stepsize, which would not require the above specific choices. While we kept in this work the already implemented and highly tested analytical approach of TRiP98, future implementation of a numerical line search method could be in principle possible and useful.

Besides the ones here presented (SD and CGFR), there are alternative algorithms for non-linear optimization, such as BFGS (9), already implemented in TRiP98, and several others not yet implemented, such as *interior-point method* (31) and *sequential quadratic programming* (32). With this work, we wanted to implement a new optimization approach based on gEUD in TRiP98, while staying as close as possible to the already implemented optimization routines. The implementation of additional algorithms, however, could be evaluated in the future.

Furthermore, in principle, the gEUD-based quadratic cost function presented in this work could be applied independently of the optimization routine, previously mentioned, or the biological dose model used. For example, an alternative method for the optimization of the biological effect is the one proposed by Wilkens and Oelfke (33).

Optimization based on gEUD has been extensively studied in the case of photon therapy, as in Schwarz et al. (18) and in Fogliata et al. (21), which used a quadratic cost function similar to ours, and also in Wu et al. (16), where a logistic cost function was used, but much less in the case of particle therapy. In fact, the gEUD-based optimization has already been partially explored only by Brüningk et al. (20) in the case of carbon ion therapy. Indeed, that study focused more on the equivalent uniform effect (EUE)-based optimization, using the approach proposed by Wilkens and Oelfke (33), comparing it also with the optimization based on RBE-weighted gEUD. Furthermore, the results shown there refer to organs with a small volume effect of a single plan. Finally, in that work, the influence of uncertainties in the volume effect parameter on the optimization outcome was investigated. Instead, in our work, we implemented a cost function with a quadratic penalty in RBE-weighted gEUD in order to maintain objectives on dose values and not on other quantities such as the EUE or NTCP. We decided to do this in order to make the new implementation an extension of the overall voxel-dose-based cost function of TRiP98. Moreover, in our work, a greater focus has been given to organs with a large volume effect, such as the parotid glands, in order to explore planning problems where the benefits of gEUD-based optimization are expected to be the largest. We also presented several treatment plans for which we compared voxel-dose-based optimization with the new gEUD-based approach. Finally, we also showed some technical details regarding the implementation of gEUD-based optimization, as well as some convergence tests in the **Supplementary Material**.

Another code, matRad (34), recently introduced the possibility to select a gEUD-based objective. It provides two options to perform biological optimization: the first one considers the biological effect-based optimization, according to Wilkens and Oelfke (33), while the second one takes into account the first implementation of RBE-weighted dose-based optimization used in TRiP98 (6). In our work instead, we employed the updated version of the RBE-weighted dose-based optimization, described in Krämer and Scholz (27) and Gemmel et al. (8), with the explicit inclusion of ∇*RBE_i_* in the minimization, a feature that is not present in (6), as detailed in Section 2.3.2, but it is somehow implicitly accounted in (33). Another difference is that in matRad the absolute minimization of gEUD is proposed, while in our work, a prescription is defined and a quadratic objective is considered. Finally, in that work, no results from gEUD-based optimization are shown.

### 4.1 Outlook

Beyond the gEUD-based optimization of healthy organs, the next step would be to optimize also the target with gEUD: the idea is to use a negative value of *a* in order to control low dose levels, combined with the use of a positive *a* value to control high dose levels, treating the target as an OAR. This idea can be formalized mathematically defining a new cost function for the target composed of two terms that are dependent on gEUD, replacing the uniform dose objective, namely,


(22) $$\begin{array}{c} \chi_{T}^{2}(\vec{N})=(w_{T}^{\min}{)}^{2}\frac{{\left(gEUD_{0}^{\min}-gEUD^{\min}(\vec{N})\right)}^{2}}{{\left(\Delta gEUD_{0}^{\min}\right)}^{2}}\theta_{gEUD}^{\min}\\ +(w_{T}^{\max}{)}^{2}\frac{{\left(gEUD_{0}^{\max}-gEUD^{\max}(\vec{N})\right)}^{2}}{{\left(\Delta gEUD_{0}^{\max}\right)}^{2}}\theta_{gEUD}^{\max} \end{array}$$

where the first term is for the minimum dose control, while the second one is for the maximum dose control, and


(23) $$\begin{array}{l} \theta_{gEUD}^{\min}=\theta (gEUD_{0}^{\min}-gEUD^{\min}(\vec{N}))=\{\begin{array}{ll} 1, & gEUD^{\min}(\vec{N})<gEUD_{0}^{\min}\\ 0, & gEUD^{\min}(\vec{N})\geq gEUD_{0}^{\min} \end{array}\\ \theta_{gEUD}^{\max}=\theta (gEUD^{\max}(\vec{N})-gEUD_{0}^{\max})=\{\begin{array}{ll} 1, & gEUD^{\max}(\vec{N})>gEUD_{0}^{\max}\\ 0, & gEUD^{\max}(\vec{N})\leq gEUD_{0}^{\max} \end{array} \end{array}$$

are Heavyside functions in order to penalize the target if the actual gEUD values are smaller or larger than the prescribed values, respectively. In principle, using two gEUD objectives with two volume parameters does allow to control both high and low doses in the target. In theory, the advantage of this approach is to relax the objectives on the target, and when combined with the gEUD-based optimization of the OARs, it should allow for further sparing of them. Obviously, this should be demonstrated in clinical cases.

Another possible future step could be to move from the gEUD-based optimization of healthy organs to a direct NTCP-based optimization. As already mentioned above, in this work, we implemented a gEUD-based optimization because this is located in the dose space, and therefore, it is sufficient to integrate an additional term in the overall cost function to take into account the volume effect during the optimization task. Therefore, this allows to choose between optimization based on gEUD or on maximum dose depending on the type of OAR considered. Furthermore, given the close link between gEUD and NTCP as seen in equations 2 and 3, minimizing gEUD means also minimizing NTCP; this is also evident from the results obtained for patient 335, where the decrease in gEUD for the parotid glands corresponds to a reduction in the corresponding NTCP. Instead, the NTCP-based optimization is located in the probability space, and it becomes necessary if we want to optimize the absolute risk of complication for an organ for which more complications are associated or if we want to minimize the probability of complications for multiple OARs. Kierkels et al. (35) proposed a method in order to consider multivariable NTCP models in treatment plan optimization in the case of photon therapy. They demonstrated the feasibility of using NTCP-based optimization in the case of head and neck cancer and compared this method with gEUD-based optimization, obtaining in both cases clinically acceptable plans with small differences. According to them, one of the advantages is that NTCP models combine multiple factors into a single objective, but at the same time, as described by Witte et al. (36), in order to use NTCP in the optimization task, it is necessary to implement a complex objective function. On the other hand, according to Wu et al. (17), one of the advantages of gEUD-based optimization over other methods, such as dose–volume-based or NTCP-based optimizations, is that it requires fewer planning parameters.

Finally, a combination of DVH-based and gEUD-based objectives may be of interest for specific OARs where DVH point constraints are commonly enforced in clinical practice.

## 5 Conclusions

In conclusion, we reported the first implementation of gEUD-based optimization in TRiP98 for carbon ion therapy, adding a new term in the cost function, in order to take into account for volume effects in the optimization task. The present implementation, coupling organ structures with RBE-weighted dose consideration, allows a strong accounting of biological effects in particle beam treatment planning. In particular, it allows to control the whole DVH shape of an OAR using a single objective, reducing different dose levels depending on the value of the chosen volume effect parameter, i.e., increasing the sparing of the organ considered. In particular, for organs with a large volume effect, it is possible to reduce their NTCP. This approach could also be extended to the target, in principle to obtain a further sparing of healthy organs. Finally, the gEUD-based optimization seems to be an excellent compromise between not taking at all into account the volume effect (voxel-dose-based optimization) and the direct minimization of NTCP (NTCP-based optimization).

## Code Availability Statement

TRiP98 full documentation, including instructions for getting a stable version of the code on request by the main developer, is available on its official webpage http://bio.gsi.de/DOCS/trip98.html.

## Data Availability Statement

The raw data supporting the conclusions of this article will be made available by the authors, without undue reservation.

## Author Contributions

MB developed the novel code implementations, performed all the calculations, analyzed the data, and wrote the first draft of the manuscript. MS and ES conceived the work and supervised it. MK guided and assisted the TRiP98 code implementations. All the authors critically read and edited the manuscript.

## Funding

This work was funded by INFN CSNV Call “MoVe IT”, Modeling and verification for ion beam treatment planning.

## Conflict of Interest

The authors declare that the research was conducted in the absence of any commercial or financial relationships that could be construed as a potential conflict of interest.

## Publisher’s Note

All claims expressed in this article are solely those of the authors and do not necessarily represent those of their affiliated organizations, or those of the publisher, the editors and the reviewers. Any product that may be evaluated in this article, or claim that may be made by its manufacturer, is not guaranteed or endorsed by the publisher.
