# Supplementary material for: Including Volume Effects in Biological Treatment Plan Optimization for Carbon Ion Therapy: Generalized Equivalent Uniform Dose-Based Objective in TRiP98
Source: Front Oncol. 2022 Mar 21;12:826414. doi: 10.3389/fonc.2022.826414 (PMC8979211; doi:10.3389/fonc.2022.826414)
Supplement: Supplementary file 1 [file DataSheet_1.pdf]

# Supplementary Material

## 1 OPTIMIZATION ALGORITHMS AND CONVERGENCE TESTS

The steepest descent (SD) approach consists in minimizing the cost function $\chi^2(\vec{N}_k)$ along the steepest descent direction $\vec{h}_k$, which is defined as the negative gradient of the cost function $\vec{g}_k$, at each iteration $k$, i.e.

$$\vec{h}_k = \vec{g}_k = -\nabla\chi^2(\vec{N}) \tag{S1}$$

while the Fletcher-Reeves variant of conjugated gradients (CGFR) method is a modification of the first one, where in this case the minimization direction $\vec{h}_k$ takes into account the previous successful iterations, so that the direction is towards the minimum of the cost function, i.e.

$$\vec{h}_k = \vec{g}_k + \gamma_{k-1}\vec{h}_{k-1} \tag{S2}$$

with

$$\gamma_{k-1} = \frac{\vec{g}_k^T \cdot \vec{g}_k}{\vec{g}_{k-1}^T \cdot \vec{g}_{k-1}} \tag{S3}$$

and therefore, in principle, CGFR converges faster with respect to SD.

CGFR and SD algorithms are tested and compared considering the same plan used for the studying of the role of the cost function parameters. Observing for example the figure S1 we can see that CGFR has a faster and more accurate convergence than SD; in fact the cost function value $\chi^2$ is lower at each iteration $k$ in the case of CGFR compared to SD (figure S1A: note that the slight increase in the gEUD-cost function component at the initial iterations is balanced by a target improvement yielding to an overall decrease of the total cost function). This implies also better results in terms of DVHs (figure S1B): in particular the target curve, in the case of CGFR, is steeper than the SD curve, i.e. the percentage of the target volume which receives doses between $90\%$ and $100\%$ is higher for CGFR. At the same time in the case of CGFR the OAR curve is lower with respect to the OAR curve for SD, i.e. the mean dose received by the OAR is lower for CGFR.

In order to test the convergence for different volume effect parameters, the previous plan is optimized using different $a$ values for the parotid gland in the case of gEUD-based optimization. Both iterative optimization algorithms are applied. As an illustrative example, in figure S2 we show the cost function values as a function of the number of iterations for two $a$ values used for the optimization of the parotid, one for $a = 1$ (red line) and one for $a > 1$ (blue line). In particular, we can see that $\chi^2$ decreases with increasing $k$, using both $a = 1$ and $a = 8$ and also using both SD (figure S2A) and CGFR (figure S2B) algorithms. We can also see that CGFR converges faster than SD for both $a$ values.

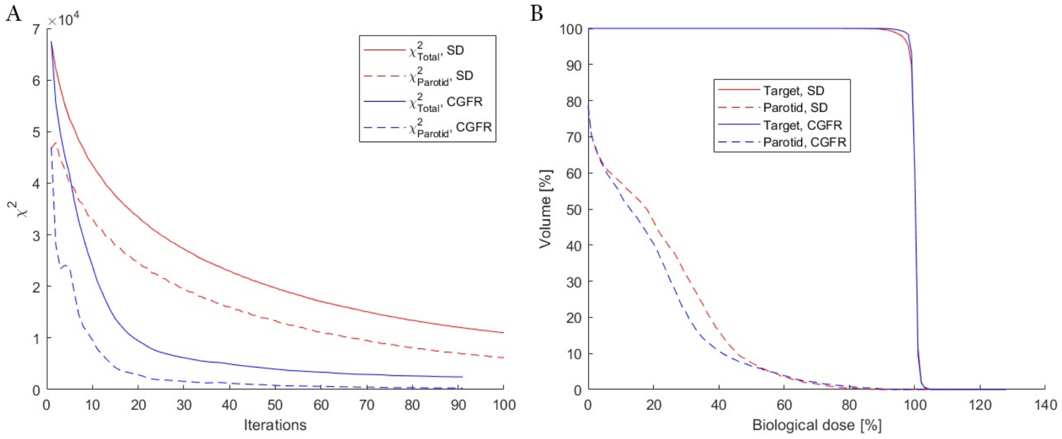


**Figure S1.** Comparison between steepest descent (SD, red curves) and Fletcher-Reeves variant of conjugated gradients (CGFR, blue curves). **(A)** Total cost function $\chi^2_{Total}$ (solid line) and the parotid component $\chi^2_{Parotid}$ of the total cost function (dashed line) as a function of number of iterations, **(B)** corresponding DVHs: target (solid line) and parotid gland (dashed line). Cost function parameters: $D_{pre} = 3.00\ Gy$, $w_T = 1$ (target); $a = 1$, $gEUD_0 = 0.50\ Gy$, $w_{OAR} = 10$ (parotid).

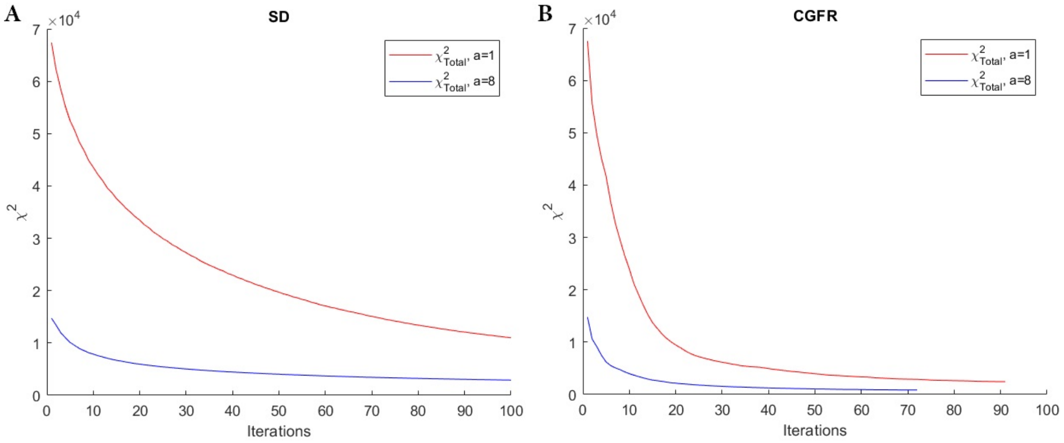


**Figure S2.** Comparison between the total cost functions as a function of number of iterations for two different values of the volume effect parameter $a$ using **(A)** SD algorithm, **(B)** CGFR algorithm. Cost function parameters: red line, $D_{pre} = 3.00\ Gy$, $w_T = 1$ (target); $a = 1$, $gEUD_0 = 0.50\ Gy$, $w_{OAR} = 10$ (parotid). Blue line, $D_{pre} = 3.00\ Gy$, $w_T = 1$ (target); $a = 8$, $gEUD_0 = 1.50\ Gy$, $w_{OAR} = 20$ (parotid).

## 2 STEPSIZE SOLUTION DETAILS

The expression obtained for the physical stepsize is

$$\mu_k^{phys} \simeq \frac{\mathrm{NUM}_{phys}}{\mathrm{DEN}_{phys}} \tag{S4}$$

where the numerator is

$$\begin{aligned}
\mathrm{NUM}_{phys} = &(w_T)^2 \sum_{i=1}^{M_T} \frac{\left(D_{pre} - \vec{d}_i^T \vec{N}_k\right)}{(\Delta D_{pre})^2} \cdot \vec{d}_i^T \vec{h}_k + \\
&+ (w_{OAR})^2 \sum_{i=1}^{M_{OAR}} \frac{\left(D_{max} - \vec{d}_i^T \vec{N}_k\right)}{(\Delta D_{max})^2} \cdot \vec{d}_i^T \vec{h}_k \cdot \theta_{D_{max}} + \\
&+ \frac{(w_{OAR})^2}{(\Delta gEUD_0)^2} \left[gEUD_0 - gEUD(D_i^{phys}(\vec{N}_k))\right] \frac{1}{M_{OAR}} \sum_{i=1}^{M_{OAR}} \vec{d}_i^T \vec{h}_k \cdot \theta_{gEUD}
\end{aligned} \tag{S5}$$

with

$$gEUD(D_i^{phys}(\vec{N}_k)) = \left(\frac{1}{M_{OAR}} \sum_{i=1}^{M_{OAR}} \left(\vec{d}_i^T \vec{N}_k\right)^a\right)^{\frac{1}{a}} \tag{S6}$$

which corresponds to the gEUD value at iteration $k$, while the denominator is

$$\begin{aligned}
\mathrm{DEN}_{phys} = &(w_T)^2 \sum_{i=1}^{N_T} \frac{\left(\vec{d}_i^T \vec{h}_k\right)^2}{(\Delta D_{pre})^2} + (w_{OAR})^2 \sum_{i=1}^{M_{OAR}} \frac{\left(\vec{d}_i^T \vec{h}_k\right)^2}{(\Delta D_{max})^2} \cdot \theta_{D_{max}} + \\
&+ \left(\frac{w_{OAR}}{\Delta gEUD_0} \frac{1}{M_{OAR}} \sum_{i=1}^{M_{OAR}} \vec{d}_i^T \vec{h}_k\right)^2 \cdot \theta_{gEUD}
\end{aligned} \tag{S7}$$

The parameters of this expression are defined in the main text, in the Materials and Methods section.

## 3 ADDITIONAL TREATMENT PLANS

A series of comparisons for a number of additional plans are reported in the following to illustrate the behavior of our method for different specific situations. The prescriptions for the plans for both voxel-dose-based and gEUD-based optimization are reported in table S1.

The treatment plan 299 is again a chordoma case, where multiple OARs with small volume effects are considered: the brainstem, the right and the left optic nerves and the chiasm. The target is irradiated with two nearly opposite fields, with (couch) angles $-104°$ and $104°$, according to the original plan.

Observing the gEUD values and the maximum doses of the OARs coming from the optimization (table S2), the dose distributions (figures S3A and S3B) and the DVHs (figure S3C) it is possible to see that the two plans are very similar, with identical results in terms of gEUD values of the OARs, and with a little improvement for the right optic nerve: in fact the DVH obtained from the gEUD-based optimization, using $a = 20$, is below that obtained by requiring a maximum dose as an objective; this corresponds to a slight decrease of the gEUD value.

Another additional treatment plan is the patient 394, again a chordoma case, which contains the spinal cord and both parotid glands in the fields direction, as OARs, i.e. a peculiar geometrical situation. The tumor is irradiated using two opposite fields, with (couch) angles $-90°$ and $90°$, according to the original plan.

For this patient, looking at the results in table S3 for the OARs, the DVHs in figure S4 and the dose distributions in figure S5, it is possible to conclude, also in this case, that the gEUD-based optimization allows to reduce the mean dose received by the parotid glands, considering $a = 1$, that corresponds to a reduction of their NTCP values (table S3 and figure S6), considering EQD2 calculation. Moreover, a slight decrease of the gEUD value for the spinal cord is obtained, using $a = 20$ (see table S3).

Finally, the treatment plan 339 contains multiple OARs with small volume effects (brainstem, spinal cord, right and left optic nerves and chiasm) and both parotid glands. This plan is an extreme geometrical situation where a parotid is proximal to the target. The target considered in this case is the GTV, according to the original plan. The tumor is irradiated using two nearly opposite fields, with (couch) angles $-90°$ and $85°$, according to the original plan.

Also for this patient, a mean dose decrease (see table S4 and figures S7 and S8), and therefore a NTCP reduction (figure S9), considering EQD2 calculation, for both parotids is achieved using gEUD-based optimization as compared to voxel-dose-based approach. However, comparing the results from the two optimization approaches, the improvement in sparing of the right parotid, due to its proximal position to the target, involves a different dose distribution in the distal area, as shown in figure S8. This did not happen in the other cases shown. At the same time there is a small improvement also for serial organs, for example the right optic nerve (see table S4), probably due to the considerable sparing of the proximal parotid.

| | Plan 299 | |
|---|---|---|
| **VOI** | **Voxel-dose-based opt.** | **gEUD-based opt.** |
| Target | $D_{pre} = 3.00\,Gy, w_T = 1$ | $D_{pre} = 3.00\,Gy, w_T = 1$ |
| Brainstem | $D_{max} = 1.50\,Gy, w_{OAR} = 1$ | $gEUD_0 = 1.30\,Gy, a = 20, w_{OAR} = 20$ |
| Right optic nerve | $D_{max} = 2.10\,Gy, w_{OAR} = 1$ | $gEUD_0 = 1.80\,Gy, a = 20, w_{OAR} = 20$ |
| Left optic nerve | $D_{max} = 2.10\,Gy, w_{OAR} = 1$ | $gEUD_0 = 1.80\,Gy, a = 20, w_{OAR} = 20$ |
| Chiasm | $D_{max} = 1.50\,Gy, w_{OAR} = 1$ | $gEUD_0 = 1.40\,Gy, a = 20, w_{OAR} = 20$ |
| | **Plan 394** | |
| **VOI** | **Voxel-dose-based opt.** | **gEUD-based opt.** |
| Target | $D_{pre} = 3.00\,Gy, w_T = 1$ | $D_{pre} = 3.00\,Gy, w_T = 1$ |
| Right parotid | $D_{max} = 2.10\,Gy, w_{OAR} = 1$ | $gEUD_0 = 1.20\,Gy, a = 1, w_{OAR} = 20$ |
| Left parotid | $D_{max} = 2.10\,Gy, w_{OAR} = 1$ | $gEUD_0 = 1.20\,Gy, a = 1, w_{OAR} = 20$ |
| Spinal cord | $D_{max} = 1.65\,Gy, w_{OAR} = 1$ | $gEUD_0 = 1.25\,Gy, a = 20, w_{OAR} = 20$ |
| | **Plan 339** | |
| **VOI** | **Voxel-dose-based opt.** | **gEUD-based opt.** |
| Target | $D_{pre} = 3.00\,Gy, w_T = 1$ | $D_{pre} = 3.00\,Gy, w_T = 1$ |
| Right parotid | $D_{max} = 2.25\,Gy, w_{OAR} = 1$ | $gEUD_0 = 0.70\,Gy, a = 1, w_{OAR} = 20$ |
| Left parotid | $D_{max} = 2.25\,Gy, w_{OAR} = 1$ | $gEUD_0 = 0.50\,Gy, a = 1, w_{OAR} = 20$ |
| Brainstem | $D_{max} = 1.65\,Gy, w_{OAR} = 1$ | $gEUD_0 = 1.20\,Gy, a = 20, w_{OAR} = 20$ |
| Spinal cord | $D_{max} = 1.65\,Gy, w_{OAR} = 1$ | $gEUD_0 = 1.20\,Gy, a = 20, w_{OAR} = 20$ |
| Right optic nerve | $D_{max} = 1.65\,Gy, w_{OAR} = 1$ | $gEUD_0 = 1.30\,Gy, a = 20, w_{OAR} = 20$ |
| Left optic nerve | $D_{max} = 1.65\,Gy, w_{OAR} = 1$ | $gEUD_0 = 1.30\,Gy, a = 20, w_{OAR} = 20$ |
| Chiasm | $D_{max} = 1.65\,Gy, w_{OAR} = 1$ | $gEUD_0 = 1.00\,Gy, a = 20, w_{OAR} = 20$ |

**Table S1.** Cost function parameters for the plans 299, 394 and 339.

| Parameter | Voxel-dose-based opt. | gEUD-based opt. |
|---|---|---|
| | **Target** | |
| $D_{min}$ | 2.00 $Gy$ | 1.97 $Gy$ |
| $D_{max}$ | 3.30 $Gy$ | 3.31 $Gy$ |
| $D_{mean}$ | 3.00 $Gy$ | 3.00 $Gy$ |
| $CI$ | 1.17 | 1.16 |
| | **Brainstem** | |
| $gEUD\ (a = 20)$ | 1.47 $Gy$ | 1.46 $Gy$ |
| $D_{max}$ | 2.15 $Gy$ | 2.00 $Gy$ |
| | **Right optic nerve** | |
| $gEUD\ (a = 20)$ | 1.97 $Gy$ | 1.81 $Gy$ |
| $D_{max}$ | 2.39 $Gy$ | 2.15 $Gy$ |
| | **Left optic nerve** | |
| $gEUD\ (a = 20)$ | 1.88 $Gy$ | 1.82 $Gy$ |
| $D_{max}$ | 2.32 $Gy$ | 2.27 $Gy$ |
| | **Chiasm** | |
| $gEUD\ (a = 20)$ | 1.54 $Gy$ | 1.48 $Gy$ |
| $D_{max}$ | 1.94 $Gy$ | 1.79 $Gy$ |

**Table S2.** gEUD values and maximum doses of the OARs obtained for the plan 299. Minimum, maximum and mean doses and conformity index ($CI =$ volume $95\%$ isodose $/$ volume $VOI$) for the target are also shown.

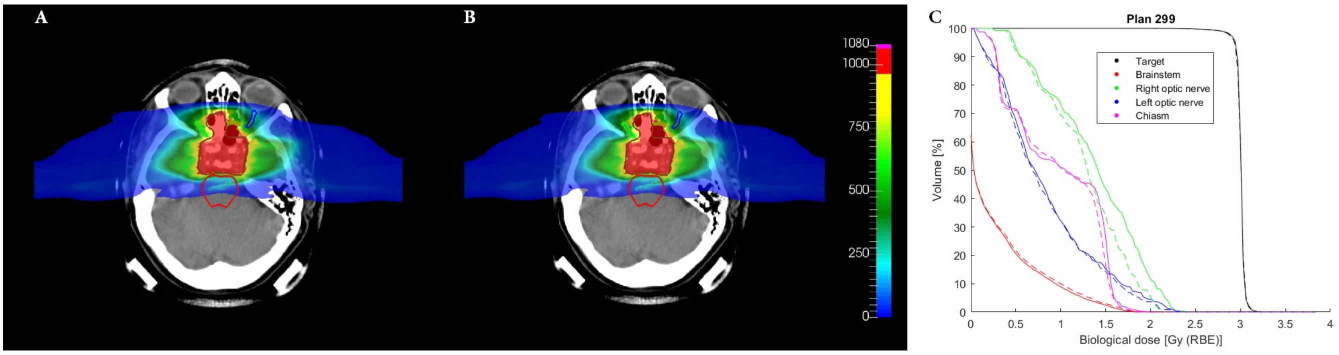


**Figure S3.** Comparison of dose distributions on a CT slice, for the patient plan 299, obtained with, **(A)** voxel-dose-based, **(B)** gEUD-based optimization. The target (brown contour), the brainstem (red contour), the right optic nerve (green contour) and the left optic nerve (blue contour) are shown. The dose levels are plotted in per mil of the prescribed dose. **(C)** Comparison of DVHs obtained with voxel-dose-based (solid line) and gEUD-based (dashed line) optimization for the patient plan 299.

| Parameter | Voxel-dose-based opt. | gEUD-based opt. |
|---|---|---|
| | **Target** | |
| $D_{min}$ | $2.58\ Gy$ | $2.33\ Gy$ |
| $D_{max}$ | $3.16\ Gy$ | $3.21\ Gy$ |
| $D_{mean}$ | $2.99\ Gy$ | $2.99\ Gy$ |
| $CI$ | $1.18$ | $1.14$ |
| | **Right parotid** | |
| $gEUD\ (a = 1)$ | $1.49\ Gy$ | $1.23\ Gy$ |
| $NTCP$ | $43.45\ \%$ | $28.58\ \%$ |
| $D_{max}$ | $2.22\ Gy$ | $2.27\ Gy$ |
| | **Left parotid** | |
| $gEUD\ (a = 1)$ | $1.45\ Gy$ | $1.23\ Gy$ |
| $NTCP$ | $41.05\ \%$ | $28.53\ \%$ |
| $D_{max}$ | $2.22\ Gy$ | $2.59\ Gy$ |
| | **Spinal cord** | |
| $gEUD\ (a = 20)$ | $1.25\ Gy$ | $1.14\ Gy$ |
| $D_{max}$ | $1.69\ Gy$ | $1.63\ Gy$ |

**Table S3.** gEUD values and maximum doses of the OARs obtained for the plan 394. In addition NTCP values (considering EQD2 calculation) for both parotids are reported. Minimum, maximum and mean doses and conformity index ($CI$ = volume $95\%$ isodose $/$ volume $VOI$) for the target are also shown.

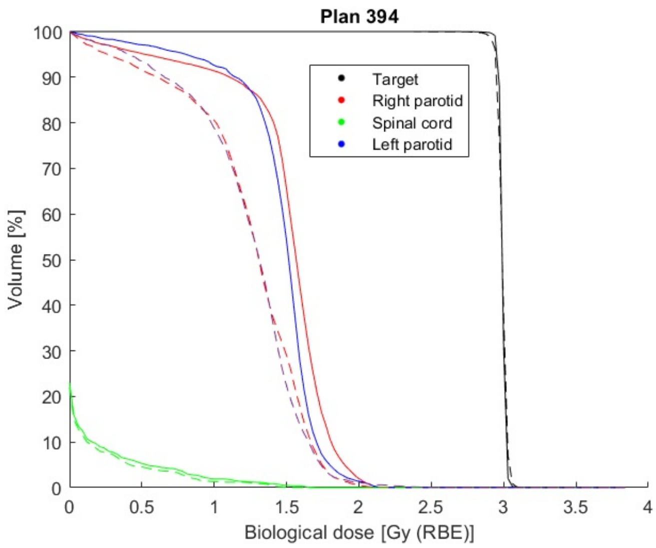


**Figure S4.** Comparison of DVHs obtained with voxel-dose-based (solid line) and gEUD-based (dashed line) optimization for the patient plan 394.

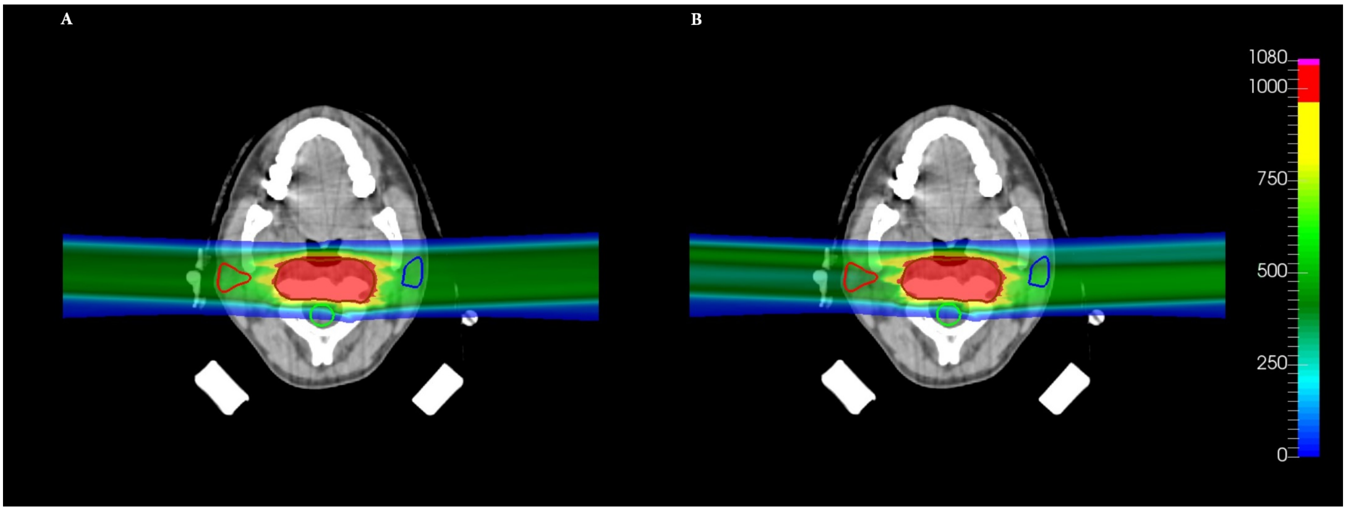


**Figure S5.** Comparison of dose distributions on a CT slice, for the patient plan 394, obtained with, **(A)** voxel-dose-based, **(B)** gEUD-based optimization. The target (brown contour), the right parotid (red contour), the left parotid (blue contour) and the spinal cord (green contour) are shown. The dose levels are plotted in per mil of the prescribed dose.

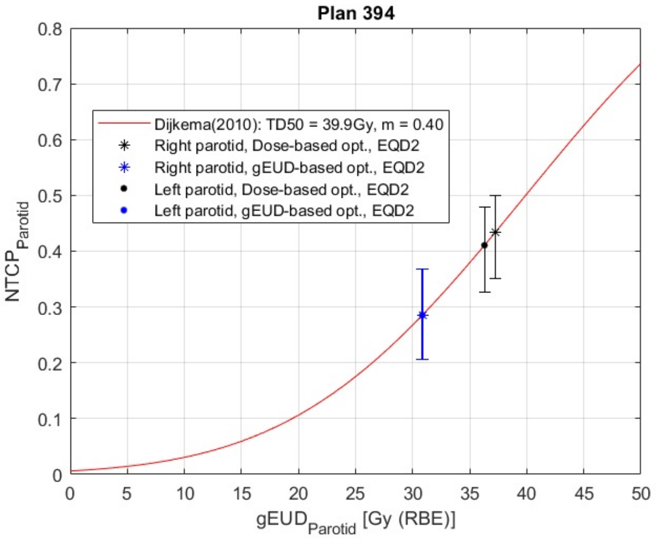


**Figure S6.** NTCP curve for the parotid glands of patient plan 394, calculated according to LKB model, using the parameters obtained by Dijkema *et al.* (2010). The error bars were calculated considering the maximum and the minimum NTCP values coming from the combination of the extreme values of the parameters $TD_{50}$ and $m$ (95% confidence intervals): $TD_{50} = 37.3\ Gy$ and $m = 0.51$ for the highest NTCP value, $TD_{50} = 42.8\ Gy$ and $m = 0.34$ for the lowest NTCP. A therapeutic plan of 20 fractions of $3\ Gy$ is considered, with EQD2 calculation.

| Parameter | Voxel-dose-based opt. | gEUD-based opt. |
|---|---|---|
| | **Target** | |
| $D_{min}$ | $2.66\ Gy$ | $2.09\ Gy$ |
| $D_{max}$ | $3.18\ Gy$ | $3.23\ Gy$ |
| $D_{mean}$ | $3.00\ Gy$ | $3.00\ Gy$ |
| $CI$ | $1.21$ | $1.20$ |
| | **Right parotid** | |
| $gEUD\ (a = 1)$ | $1.15\ Gy$ | $0.73\ Gy$ |
| $NTCP$ | $24.20\%$ | $8.70\%$ |
| $D_{max}$ | $2.53\ Gy$ | $2.93\ Gy$ |
| | **Left parotid** | |
| $gEUD\ (a = 1)$ | $0.60Gy$ | $0.52\ Gy$ |
| $NTCP$ | $5.97\%$ | $4.56\ \%$ |
| $D_{max}$ | $1.47\ Gy$ | $1.78\ Gy$ |
| | **Brainstem** | |
| $gEUD\ (a = 20)$ | $1.25\ Gy$ | $1.16\ Gy$ |
| $D_{max}$ | $1.67\ Gy$ | $1.62\ Gy$ |
| | **Spinal cord** | |
| $gEUD\ (a = 20)$ | $0.02\ Gy$ | $0.01\ Gy$ |
| $D_{max}$ | $0.03\ Gy$ | $0.02\ Gy$ |
| | **Right optic nerve** | |
| $gEUD\ (a = 20)$ | $1.31\ Gy$ | $1.05\ Gy$ |
| $D_{max}$ | $1.66\ Gy$ | $1.33\ Gy$ |
| | **Left optic nerve** | |
| $gEUD\ (a = 20)$ | $1.35\ Gy$ | $1.28\ Gy$ |
| $D_{max}$ | $1.66\ Gy$ | $1.61\ Gy$ |
| | **Chiasm** | |
| $gEUD\ (a = 20)$ | $0.96\ Gy$ | $0.97\ Gy$ |
| $D_{max}$ | $1.27\ Gy$ | $1.27\ Gy$ |

**Table S4.** gEUD values and maximum doses of the OARs obtained for the plan 339. In addition NTCP values for both parotids are reported. Minimum, maximum and mean doses and conformity index ($CI =$ volume $95\%$ isodose / volume $VOI$) for the target are also shown.

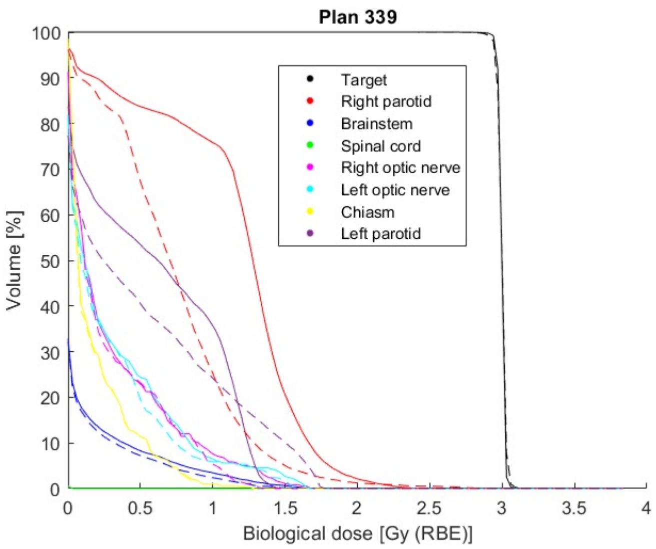


**Figure S7.** Comparison of DVHs obtained with voxel-dose-based (solid line) and gEUD-based (dashed line) optimization for the patient plan 339.

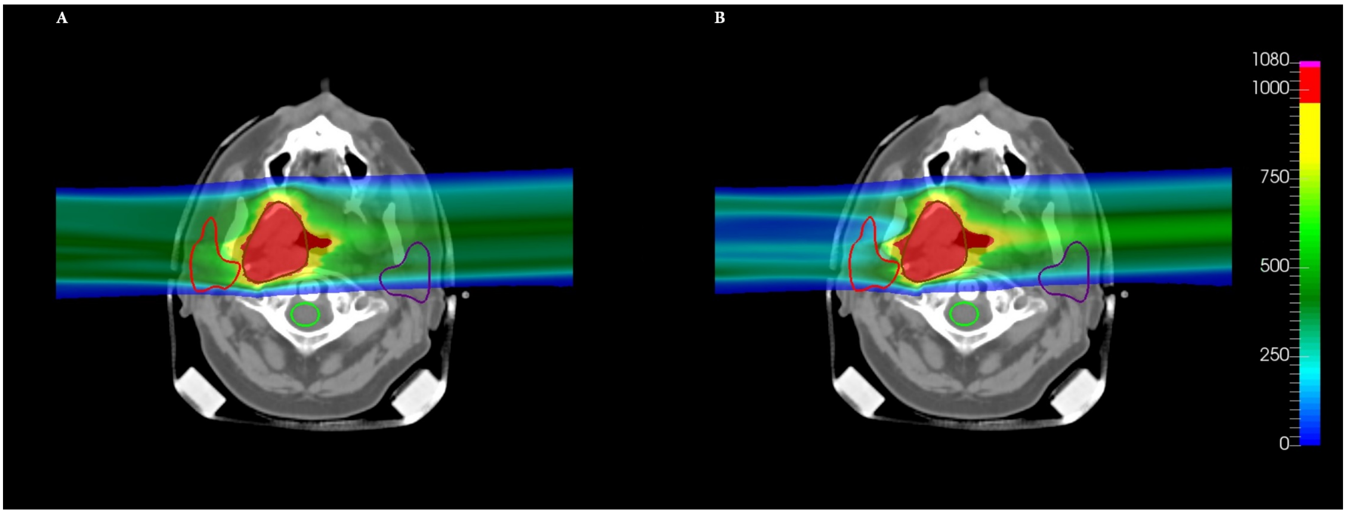


**Figure S8.** Comparison of dose distributions on a CT slice, for the patient plan 339, obtained with, **(A)** voxel-dose-based, **(B)** gEUD-based optimization. The target (brown contour), the right parotid (red contour), the left parotid (violet contour) and spinal cord (green contour) are shown. The dose levels are plotted in per mil of the prescribed dose.

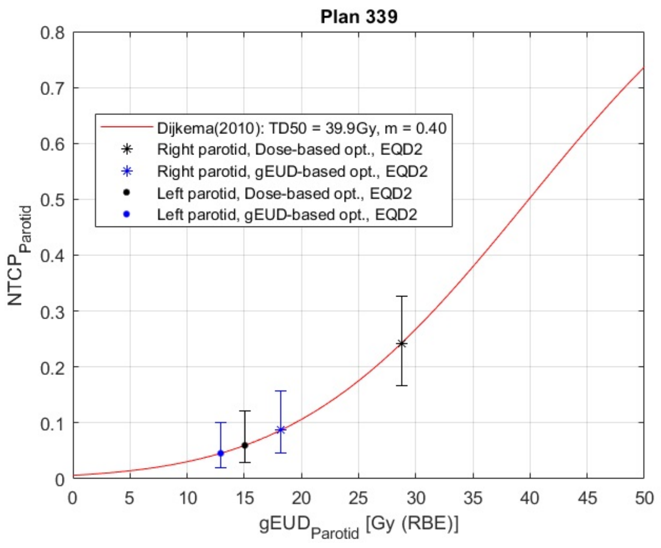


**Figure S9.** NTCP curve for the parotid glands of patient plan 339, calculated according to LKB model, using the parameters obtained by Dijkema *et al.* (2010). The error bars were calculated considering the maximum and the minimum NTCP values coming from the combination of the extreme values of the parameters $TD_{50}$ and $m$ ($95\%$ confidence intervals): $TD_{50} = 37.3\ Gy$ and $m = 0.51$ for the highest NTCP value, $TD_{50} = 42.8\ Gy$ and $m = 0.34$ for the lowest NTCP. A therapeutic plan of $20$ fractions of $3\ Gy$ is considered, with EQD2 calculation.
